# Discrete genetic subtypes and tumor microenvironment signatures correlate with peripheral T-cell lymphoma outcomes

**DOI:** 10.1038/s41375-025-02563-0

**Published:** 2025-03-31

**Authors:** Yasuhito Suehara, Kana Sakamoto, Manabu Fujisawa, Kota Fukumoto, Yoshiaki Abe, Kenichi Makishima, Sakurako Suma, Tatsuhiro Sakamoto, Keiichiro Hattori, Takeshi Sugio, Koji Kato, Koichi Akashi, Kosei Matsue, Kentaro Narita, Kengo Takeuchi, Joaquim Carreras, Naoya Nakamura, Kenichi Chiba, Yuichi Shiraishi, Satoru Miyano, Seishi Ogawa, Shigeru Chiba, Mamiko Sakata-Yanagimoto

**Affiliations:** 1 Department of Hematology, University of Tsukuba Hospital, Tsukuba, Japan; 2 Department of Hematology, Institute of Medicine, University of Tsukuba, Tsukuba, Japan; 3 Pathology Project for Molecular Targets, The Cancer Institute, Japanese Foundation for Cancer Research, Tokyo, Japan; 4 Division of Pathology, Cancer Institute, Japanese Foundation for Cancer Research, Tokyo, Japan; 5 Centre for Lymphoid Cancer department, BC Cancer, Vancouver, BC Canada; 6 Department of Medicine and Biosystemic Science, Kyushu University Graduate School of Medical Science, Fukuoka, Japan; 7 Department of Medicine, Division of Oncology, Stanford University, Stanford, CA USA; 8 Division of Hematology/Oncology, Department of Internal Medicine, Kameda Medical Center, Kamogawa, Japan; 9 Department of Pathology, Cancer Institute Hospital, Japanese Foundation for Cancer Research, Tokyo, Japan; 10 Department of Pathology, Tokai University School of Medicine, Isehara, Japan; 11 Department of Genome Analysis Platform Development, National Cancer Center Research Institute, Tokyo, Japan; 12 M&D Data Science Center, Tokyo Medical and Dental University, Tokyo, Japan; 13 Department of Pathology and Tumor Biology, Kyoto University, Kyoto, Japan; 14 Institute for the Advanced Study of Human Biology (WPI-ASHBi), Kyoto University, Kyoto, Japan; 15 Division of Advanced Hemato-Oncology, Transborder Medical Research Center, University of Tsukuba, Tsukuba, Japan

**Keywords:** T-cell lymphoma, Cancer genomics

## Abstract

Peripheral T-cell lymphoma (PTCL) exhibits a diverse clinical spectrum, necessitating methods to categorize patients based on genomic abnormalities or tumor microenvironment (TME) profiles. We conducted an integrative multiomics study in 129 PTCL patients, performing whole-exome sequencing and identifying three genetic subtypes: C1, C2, and C3. C2 was characterized by loss of tumor suppressor genes and chromosomal instability, while C1 and C3 shared T follicular helper (TFH)-related genomic alterations, with C3 also showing a high incidence of *IDH2* mutations and chromosome 5 gain. Compared to C1, survival was significantly worse in C2 (HR 2.52; 95% CI, 1.37–4.63) and C3 (HR 2.14; 95% CI, 1.17–3.89). We also estimated the proportions of immune cell fractions from the bulk RNA sequencing data using CIBERSORTx and classified TME signatures into the following hierarchical clusters: TME1 (characterized by increased B and TFH cells), TME2 (macrophages), and TME3 (activated mast cells). TME2 was associated with shorter survival (HR 3.4; 95% CI, 1.6–7.5) and was more frequent in C2 (64.3%) than in C1 (7.7%), whereas C1 had more TME3 signatures (80.8% vs. 28.6%). These findings highlight a significant relationship between genetic subtypes and TME signatures in PTCL, with important implications for clinical prognosis.

## Introduction

Nodal T-follicular helper (TFH) lymphoma (nTFHL) is a subset of mature T-cell lymphomas in which tumor cells share similar gene expression patterns and immunohistochemical phenotypes with normal TFH cells. Angioimmunoblastic T-cell lymphoma (AITL) is the most common histological subtype of nTFHL, with diverse immune cell infiltration, follicular dendritic cell meshwork proliferation, and prominent high endothelial venules (HEVs). Additionally, characteristic mutation burdens are a hallmark of nTFHL, as massive parallel sequencing revealed abnormalities in the components of epigenetic regulators, the T-cell receptor (TCR) signaling pathway, and *RHOA* Gly17Val [1–8]. Among the epigenetic regulators, *TET2* is the most frequently mutated (up to 80% of cases), followed by *DNMT3A* (20–30%). These mutations are putatively acquired in bone marrow-resident hematopoietic stem cells during aging [9], as they are found during clonal hematopoiesis (CH), suggesting that nTFHL arises from CH [10–12]. Notably, infiltrating immune and tumor cells harbor *TET2* mutations, possibly affecting the tumor microenvironment (TME) in AITL [10, 13, 14]. *IDH2* Arg172, another driver mutation in epigenetic regulation, is observed in 20–30% of AITL cases and rarely in other nTFHL subtypes [15, 16], whereas the *RHOA* Gly17Val mutation, specific for nTFHL, occurs in ~50–70% of cases [4–6]. This mutation enhances the interaction between RHOA (a small GTPase) and VAV1 (an adapter molecule in the TCR signaling pathway), thereby intensifying TCR signal transduction [17]. Conversely, alterations in TCR signaling pathway components, such as *PLCG1*, *CD28*, and *VAV1*, are common in several peripheral T-cell lymphoma (PTCL) subtypes [5, 17–21].

The survival prognosis of patients with AITL is generally poor, with a median overall survival of 3 years [22]. However, approximately one-third of patients with AITL who achieve event-free survival at 24 months show favorable outcomes, even with high scores on the international prognostic index [22]. Attempts to stratify the prognosis of AITL based on individual single-gene alterations showed limited success [15, 23–25]. Recently, a genetic subtype of PTCL-not-other specified (NOS) with loss of tumor suppressor activity and aneuploidy was associated with a worse prognosis [26, 27]. Therefore, we examined whether the exploration of genetic subtypes integrating driver events (including single-nucleotide variants, structural variants, and copy number alterations) could provide insights into the heterogeneity of AITL and the prognostic impact of the genetic subtypes.

## Methods

### Patients and pathological review

A total of 129 patients with PTCL (94 with nTFHL and 35 with PTCL-NOS), diagnosed between 1995 and 2018 with extracted DNA from tumor samples of sufficient quality for whole exome sequencing (WES) were included in this study (Fig. S1a). The cohort consists of 129 cases from three sources: UTH (University of Tsukuba Hospital)(*n* = 37, 28.7%), KMC (Kameda Medical Center) (*n* = 67, 51.9%), and the others (*n* = 25, 19.4%). The subclassification of nTFHL was as follows: 85 AITL, two follicular T-cell lymphomas (FTCL), six nodal PTCL with TFH phenotypes, and one unclassifiable TFH lymphoma, for which the subclassification could not be determined conclusively. Twenty-four of the 35 PTCL-NOS specimens were pathologically reviewed and evaluated for six TFH markers (ICOS, PD1, CD10, BCL6, CXCR5, and CXCL13) to exclude nTFHL and were designated as PTCL-NOS (WHO 2016) [28]. The remaining 11 unreviewed PTCL-NOS cases were denoted as PTCL-NOS (WHO 2008) [29]. In addition to the WES cohort described above, a validation cohort of 48 PTCL cases (24 with AITL, 3 with FTCL, and 21 with PTCL-NOS) for which survival and cytogenetic data were available was evaluated for the prognostic impact of arm-level CNA derived from WES (Fig. S1b).

### Ethics approval and consent to participate

This study was approved by the institutional review board of each institution, including UTH (H24-75), and was conducted in accordance with the Declaration of Helsinki. Informed consent was obtained from all patients enrolled in the study.

### Whole exome sequencing and analysis

Genomic DNA was extracted from frozen samples (n = 106), formalin-fixed paraffin-embedded (FFPE) samples (*n* = 23), and paired buccal mucosa samples (*n* = 18) using the QIAamp DNA Mini kit (QIAGEN) or QIAamp DNA FFPE tissue kit (QIAGEN). DNA quality was examined using TapeStation (Agilent). Exome enrichment was performed using a SureSelect Human All Exon v7 kit (Agilent). Libraries were subjected to high-throughput sequencing using an Illumina HiSeq platform. Subsequently, the obtained sequencing data were analyzed using the Genomon pipeline 2.6.2 (https://genomon-project.github.io/GenomonPagesR/) to identify mutations and structural variants (SVs). Details are described in the supplementary appendix.

### Genetic subtyping using NMF

A total of 34 genetic drivers recurrently observed in more than four cases were integrated using non-negative matrix factorization (NMF) consensus clustering. All genomic alterations were represented as 1 if present and 0 if absent. NMF was applied to the gene-sample matrix (Table S1). The cluster number of two to eight was considered, with three being adopted to maximize the cophenetic coefficient (Fig. S2). NMF was performed using the Brunet algorithm with 30 runs in the R nmf package v.0.23.0 [30].

### Estimation of TME signature

We estimated the proportion of immune cell fractions from bulk RNA sequencing data using CIBERSORTx [31]. Details of the bulk RNA sequencing methods are described in the supplementary appendix. We classified TME signatures using a hierarchical clustering method (function hclust of the R stats package v.3.6.2).

### Gene set enrichment variation analysis

To estimate the classification of PTCL based on gene expression profiling, we performed gene set enrichment analysis using R GSVA package v.1.34.0 on normalized count data from RNAseq. The analyzed gene sets are shown in Table S2.

### Survival and statistical analysis

Fisher’s exact test and *t*-test were used for categorical and continuous variables, respectively, unless otherwise noted. Multiple comparisons were corrected for false discovery using the Benjamini-Hochberg method. For survival analysis, we selected 109 patients whose samples were obtained before treatment and whose clinical information was available. The *p* values for survival were calculated using the Cox proportional hazards test. All statistical analyses were performed using R version 3.6.3.

## Results

### Mutations, focal CNAs, and SVs

**Fig. 1:**
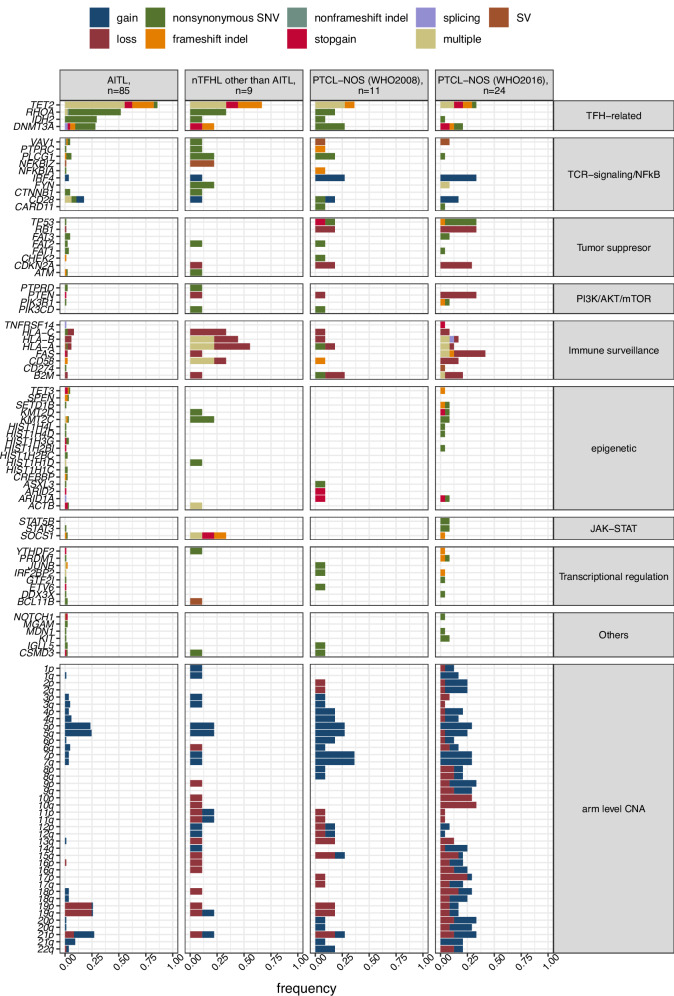
Recurrent gene alteration and arm-level copy number alterations across pathological diagnoses. Frequencies of each genomic alteration (color-coded by type) are displayed and categorized into pathway groups. SNV single nucleotide variant, SV structural variant, CNA copy number alteration, AITL angioimmunoblastic T-cell lymphoma, nTFHL nodal T-follicular helper lymphoma, PTCL-NOS peripheral T-cell lymphoma, not otherwise specified.

We performed WES on samples collected from 129 patients with PTCL (94 nTFHL [85 AITL, two FTCL, six nodal PTCL with TFH phenotypes, and one unclassifiable TFH lymphoma] and 35 PTCL-NOS) (Fig. S1a). The median mean sequencing depth for WES was 132 (interquartile range; 117.1–151.4) (Fig. S3). A median of 23 mutations (0.477 mutations/Mb) was identified. The specimen status, FFPE, and freshly frozen samples did not affect the number of SNVs (Fig. S4). We applied MutSig2CV [32] to exclude passenger mutations such as *TTN*. Nine mutations (*TET2*, *RHOA*, *DNMT3A*, *IDH2*, *TP53*, *CD28*, *PLCG1*, *CTNNB1*, and *HLA-A*) were identified as significant driver mutations using MutSig2CV (Table S3). In addition, mutations previously identified as recurrent mutations in PTCL and other lymphomas were estimated to be driver mutations (Table S4). In summary, we identified 98 recurrent driver mutations in our study cohort, with a median of four driver mutations (range: 0–18) in each sample. We confirmed the known recurrent SNVs in nTFHL and PTCL-NOS at frequencies comparable to the previous literature: TFH-related *TET2*, *RHOA*, *IDH2*, and *DNMT3A*; TCR-signaling *CD28*, *PLCG1*, and *VAV1*; tumor suppressor *TP53*; PI3K/AKT/mTOR *PIK3CD* and *PI3KR1*; immune surveillance *HLA-A*, *HLA-B*, *HLA-C*, *B2M*, and *CD58*; epigenetic modifiers *TET3*, *KMT2C*, *KMT2D*; JAK-STAT *SOCS1*, *STAT3*, and *STAT5B*; and transcriptional regulation *YTHDF2*, *PRDM1*, *JUNB*, and *DDX3X* (Fig. 1). Notably, we identified previously unrecognized recurrent mutations in histone genes (*HIST1H1C*, *HISTIH1D, HIST1H2BC, HIST1H2BI, HIST1H3G, HIST1H4D*, and *HIST1H4L*).

*TET2* mutations were most frequently detected in 91 cases, with 85.8%, 66.7%, and 34.3% of AITL, nTFHL other than AITL, and PTCL-NOS, respectively (AITL vs. PTCL-NOS, adjusted *P* value 1.56 × 10^−7^; Figs. 1, S5, S6, and S7). Fifty-five cases (59% of *TET2*-mutated cases) carried two or more *TET2* mutations as previously described [33]. Notably, among double *TET2* mutated cases, missense-missense, missense-truncating, and truncating-truncating combinations comprised only 6.5%, 37%, and 56.5% (3/46, 17/46, and 26/46) of all combinations, respectively (Fig. S6), as was previously described for myeloid neoplasms [34]. These findings suggest a potential advantage of truncating *TET2* mutations over missense mutations in the growth of hematological malignancies.

Using GISTIC2 analysis, twenty-five focal copy number changes were found to be significant (Fig. S8). The loss of regions spanning the *TRA*, *TRB*, and *TRG* loci was most significant, probably due to *TCR* rearrangement. *CD28* gain, *IRF4* gain, and *CDKN2A* losses were significant focal CNAs as previously described [26, 35, 36]. Among the ten detected *CDKN2A* losses, four were heterozygous, and five were homozygous, with the remaining losses being difficult to determine with respect to ploidy. Gene expression levels of *CDKN2A* were lower in samples with homozygous *CDKN2A* loss than in those with wild type, whereas heterozygous *CDKN2A* loss was not significantly associated with expression levels (*p* < 0.05; Fig. S9). In addition, we deemed losses of *B2M*, *CD58*, *PTEN*, *FAS*, *RB1*, and *HLA class 1* as driver CNA candidates [26, 36].

A total of 12 SVs were identified, including 3’- untranslated region (UTR) disruptions of *CD274* (Fig. S10). Remarkably, we identified three SVs of *NFKBIZ* that have not been previously reported as recurrently altered genes in nTFHL (Fig. S10). All were 3’-UTR disruptions, possibly damaging regulatory structures and activating the NF-kB pathway, as was previously described in adult T-cell leukemia/lymphoma [37].

AITL exhibited dysregulation of TFH-related genes at significantly higher frequencies than PTCL-NOS, whereas the PTCL-NOS mutational burden was primarily carried by the tumor suppressor, PI3K/AKT/mTOR, and immune surveillance pathways at significantly higher frequencies than in AITL (Fig. S11).

### Arm-level CNAs

We identified recurrent 11 arm-level CNAs in 129 patients as follows: gain of 1q, 4q, 5p, 5q, 7p, 7q, 21p, and 21q and loss of 10q, 19p, and 19q (Table S5). Arm-level CNAs, including non-recurrent CNAs, were often observed in PTCL-NOS but rarely in AITL (median arm-level CNAs per sample: PTCL-NOS vs. AITL, four vs. one; adjusted *p* = 2.5 × 10^−6^). Notably, the frequencies of gains on chromosomes (Chr)7 and 10q loss were considerably higher in PTCL-NOS (11 and eight of 35 each) than in AITL (three and zero of 85 each, adjusted *p* = 8.4 × 10^−5^ and 2.0 × 10^−5^). Gains on Chr5 (*n* = 20, 23.5%) and Chr21p (*n* = 16, 18.8%), were the prevalent events in AITL as previously described [36].

### Exclusivity and co-occurrence of driver genetic alterations

**Fig. 2:**
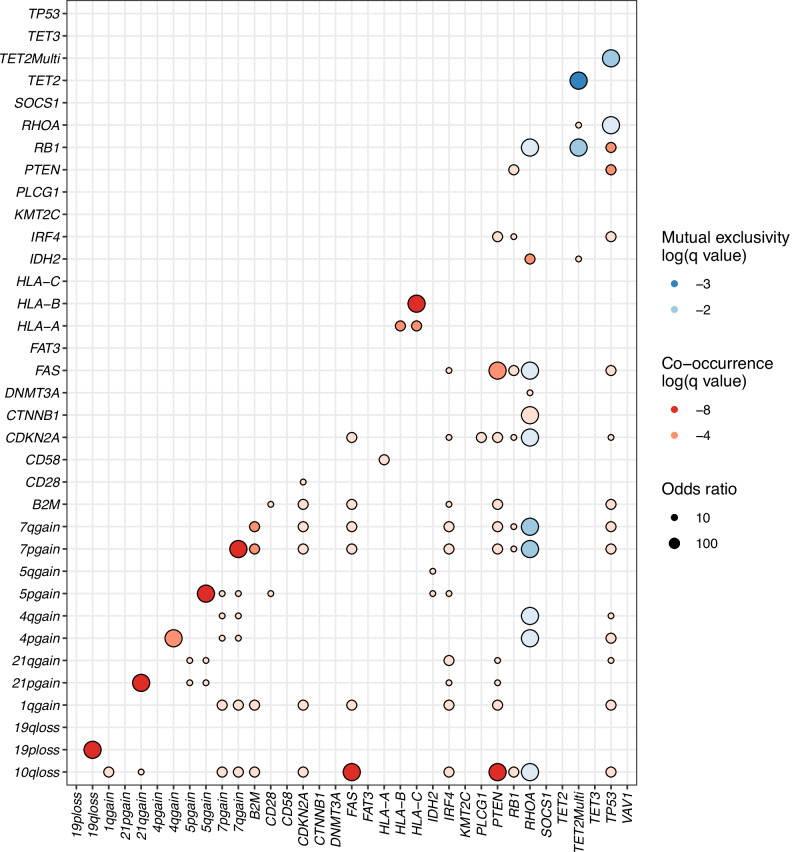
Exclusivity and co-occurrence of genetic alterations identified in nTFHL and PTCL-NOS. The hazard ratio is represented by the size of the circles. Q value is equivalent to the adjusted *p* value. *Q* values are represented by color gradient. TET2Multi, multiple *TET2* mutations.

We analyzed the patterns of exclusivity and co-occurrence of frequent driver genomic alterations. Specifically, single and multiple *TET2* mutations were analyzed separately, hypothesizing the presence of a distinct signature derived from the gene dosage effect of *TET2* as was observed in chronic myelomonocytic leukemia [34]. *RHOA* mutations were exclusive to *TP53* mutations, loss of *CDKN2A*, *FAS*, *RB1*, and several arm-level CNAs (Chr7, 4, and 10q) as previously described (Fig. 2). Interestingly, multiple *TET2* mutations, rather than one, were exclusive to the aforementioned tumor suppressor genes and arm-level CNAs. This co-occurrence of *RHOA*, *DNMT3A*, and *IDH2* is consistent with previous reports. We also confirmed the pairwise co-occurrence of *IDH2* mutations, Chr5 gain, and 21p gain, as indicated previously [36]. Remarkably, similar to the pattern of exclusivity, multiple *TET2* mutations significantly co-occurred with *RHOA* and *IDH2* mutations (Fig. 2, adjusted *p* = 0.015 and 0.011, respectively).

### Clonal structure of TFH-related gene mutations

**Fig. 3:**
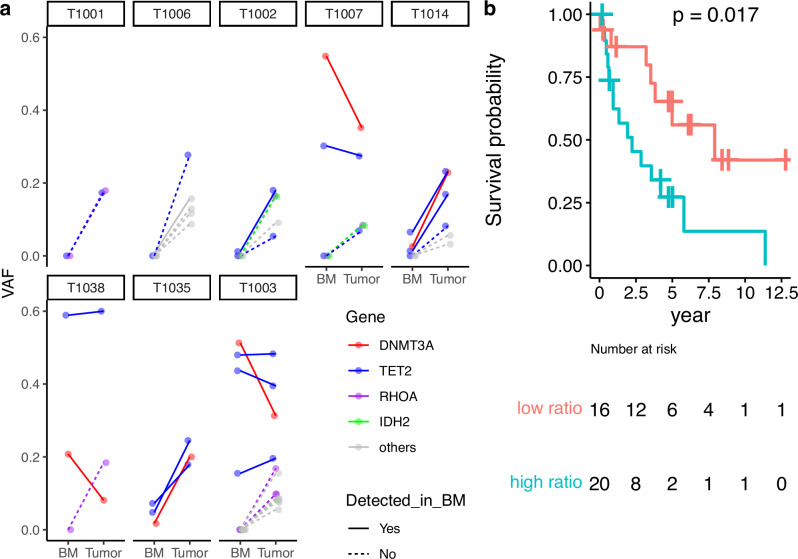
Clonal structure of TFH-related gene mutations. **a** Eight cases harboring *TET2* mutations with available paired bone marrow. If the mutation was not detected in the paired bone marrow, a dotted line is used. **b** Overall survival according to the ratio of variant allele frequencies between *TET2* and *RHOA* mutations.

These different characteristics of single and multiple *TET2* mutations prompted us to analyze the clonal structure focusing mainly on *TET2* mutations. By comparing the VAFs of mutations between tumors and paired bone marrow samples without obvious tumor infiltration in eight *TET2* mutation cases, three patterns of *TET2* mutation configuration were observed, consistent with the previous study [10, 12] (Fig. 3a). First, *TET2* mutations are exclusively acquired in tumor cells, or, if initially acquired in hematopoietic stem cells, they are restricted to differentiated progeny that give rise to tumor cells, with no detectable mutation in other non-tumor cells (T1001 and T1006 in Fig. 3a). Second, *TET2*-CH occurs first, and second-hit *TET2* mutations are acquired additionally in the tumor cells as a major clone or subclone (T1002, T1007, and T1014 in Fig. 3a). Third, there is originally one or more *TET2*-CH clones (or a single clone with biallelic *TET2* mutations), but no new *TET2* mutations are acquired in the tumor cells (T1003, T1035, and T1038 in Fig. 3a). Furthermore, the impact of *TET2* mutations on non-tumor cells was assessed by calculating the ratio of VAFs between *TET2* and *RHOA* mutations as an indicator of the expansion of *TET2*-mutated non-tumor cells. The median ratio was 1.55 and using this as a cutoff to define the high- and low-ratio groups, we found that the high-ratio group had a significantly worse prognosis than the low-ratio group (*p* = 0.017; Fig. 3b).

### Molecular subtypes of nTFHL and PTCL-NOS

**Fig. 4:**
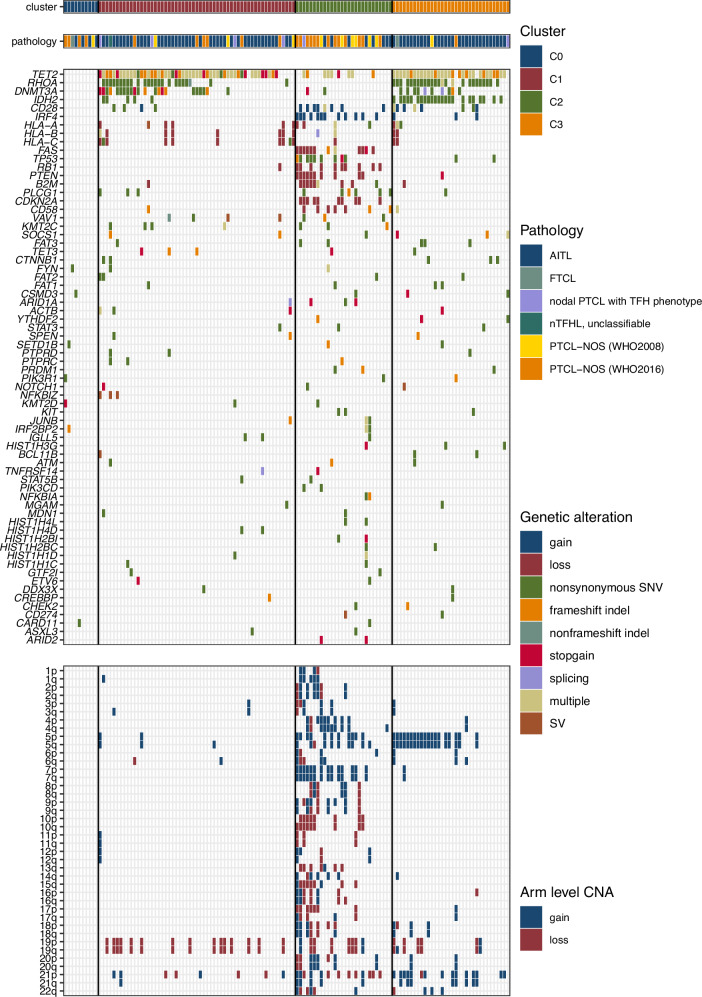
Genetic subtypes of nTFHL and PTCL-NOS. The columns represent the patients (*n* = 129) in this study. Three genetic subtypes were identified in the cohort, which are denoted as clusters 1–3; cluster 0 represents ten cases that lacked recurrent genetic abnormalities. AITL angioimmunoblastic T-cell lymphoma, FTCL follicular T-cell lymphoma, nPTCLwithTFH nodal peripheral T-cell lymphoma with T follicular helper phenotype, TFHtype_unclassifiable T-follicular helper lymphoma unclassifiable, PTCL-NOS (WHO2008) peripheral T-cell lymphoma not otherwise specified based on WHO 2008 classification, PTCL-NOS (WHO2016) peripheral T-cell lymphoma not otherwise specified based on WHO 2016 classification.

Next, we performed NMF clustering analysis to classify PTCL according to genetic alterations. We identified three genetic subtypes (clusters 1–3) within our cohort, except for 10 cases that lacked recurrent genetic abnormalities, which were denoted as cluster 0 (Figs. 4 and S2). Clusters 1 and 3 (C1 and C3; *n* = 57 and 34, respectively) mostly included nTFHL cases (84% and 94%, respectively), whereas nTFHL pathology contributed to 32% of cluster 2 (C2) cases. Five of the nine nTFHL other than AITL were C1, although the remaining four were distributed evenly across the other clusters (*n* = 1, 1, and 2 for C0, C2, and C3, respectively).

**Fig. 5:**
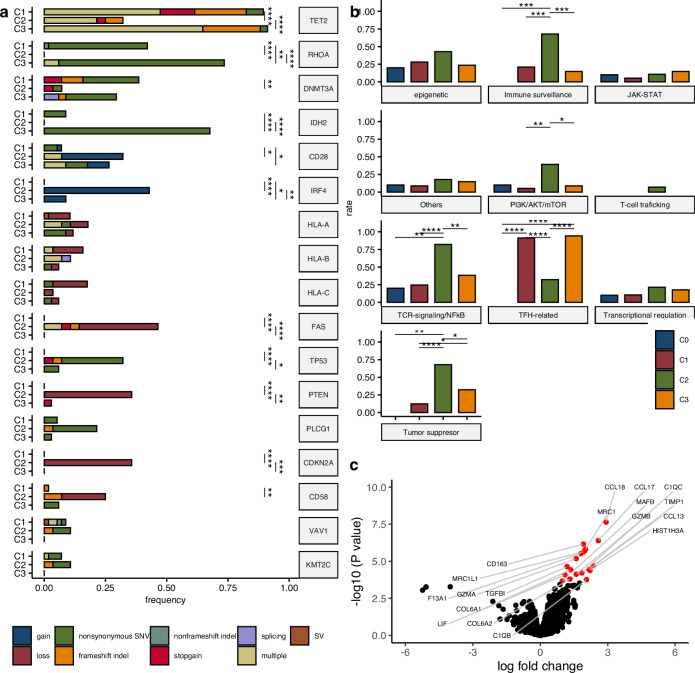
Features of genetic subtypes. **a** Difference of single gene level alterations across genetic subtypes. *, **, ***, **** represent adjusted *p* < 0.05, 0.01, 0.001, or 0.0001, respectively. **b** Frequencies of pathway alterations across the genetic subtypes. *, **, ***, **** represent adjusted *p* < 0.05, 0.01, 0.001, or 0.0001, respectively. **c** Differential gene expression analysis of C3-AITL and C1-AITL. Dots of significantly different genes are indicated in red (adjusted *p* values).

C1 and C3 shared TFH-related genomic alterations; however, C3 had significantly higher frequencies of 5p gain (55.9% vs. 3.5%), 5q gain (55.9% vs. 5.3%), 21p gain (41.2% vs. 7.0%), 21q gain (23.5% vs. 1.8%), and mutations in *RHOA* (73.5% vs 42.1%), *IDH2* (67.6% vs 8.77%), and *CD28* (26.5% vs 7.02%, Fig. 5a). The number of driver mutations was highest in C3 (Fig. S12).

Notably, 15.8% (9/57) of C1 cases were PTCL-NOS. Among the 9 PTCL-NOS cases in C1, cytotoxic markers (defined as positivity for TIA-1, granzyme B, or perforin) were positive in 6 cases, while the remaining 3 cases were not evaluated (Figs. 4 and S13). In the overall PTCL-NOS cohort, including the 6 cytotoxic-positive C1 cases, a total of 12 cases were cytotoxic marker-positive. The remaining cases were distributed across clusters as follows: 2 cases in C0 (17%), 3 cases in C2 (25%), and 1 case in C3 (8%). Specifically, among the 6 CD8+/EBER + EBV-positive nodal cytotoxic T-cell lymphoma cases, 4 (66%) were assigned to C1, with 1 case each in C0 and C3. These findings suggest that cases exhibiting a cytotoxic phenotype may share genomic abnormalities characteristic of C1. The enrichment of Epstein–Barr virus (EBV)-positive cytotoxic T-cell lymphoma in C1 possibly reflects the recurrent *TET2* and *DNMT3A* mutations in this subtype [38].

C2 was characterized by a higher tumor mutational burden and arm-level CNAs than the other subtypes (Figs. S14, S15), corresponding to the previously described GATA3-PTCL subtype (Figs. S16, S17, and S18) [26, 36]. However, 32% (*n* = 9/28) of cases in C2 are nTFHL, which share similar genomic abnormalities despite differing immunophenotypic characteristics. Notably, all nTFHL cases classified as C2 meet the diagnostic criteria for nTFHL as defined by immunohistochemistry (IHC), and gene set variation analysis (GSVA) confirmed the expression of TFH-associated gene sets in three C2-AITL cases (Figs. S16, S19).

The frequencies of genomic alterations involved in TCR-signaling/NF kappa-B, immune surveillance, PI3K/AKT/mTOR, and tumor suppressor were significantly higher in C2 than in the other subtypes (Fig. 5b). In addition, *IRF4* gain was enriched in C2, although this was not identified in previous studies of PTCL-NOS with aneuploidy and altered tumor suppressor genes [26, 36]. *MYC* expression was higher in patients with *IRF4* gain than in those without (Fig. S20), consistent with previous reports that *IRF4* overexpression caused increased *MYC* promoter activity in a PTCL cell line [35].

We then performed differential gene expression analysis between C3-AITL (*n* = 14) and C1-AITL (*n* = 23) to characterize the differences between these two major AITL genetic subgroups. Intriguingly, C3-AITL cells exhibited significant upregulation of genes encoding surface marker molecules characteristic of M2-macrophages, such as *CD163*, *MRC1L1*, *CCL17*, and *CCL18*, compared with C1-AITL cells (Fig. 5c). Subsequent pathway and process enrichment analyses using Metascape [39] revealed that genes involved in the IL-4 and IL-13 signaling pathways were enriched in C3-AITL cells (Fig. S21).

### Immunohistochemical features of genetic subtypes

Immunohistochemical features of genetic subtypes were analyzed in patients with more than 5 of 6 evaluable TFH markers (*n* = 66). C0-AITL cases meeting this criterion were limited to two cases and were therefore excluded from the genetic subtype analysis in AITL. CD10 was less frequently positive (51%) than the other five TFH markers (86–92%) in AITL, while the frequency of CD10 was lower in nTFHL other than AITL (22%) and completely absent in PTCL-NOS, indicating that CD10 was, albeit not significantly, the most specific and least sensitive marker for AITL subtype as described previously (adjusted *p* = 0.23 and 0.08 for AITL vs. nTFHL other than AITL and AITL vs. CD4-positive PTCL-NOS, respectively, Fig. S22). The number of expressed TFH markers was lower in C2-AITL than in C1-AITL and C3-AITL (median positive TFH markers: 5, 2.5, and 6 in C1, C2, and C3, respectively; *p* = 0.017 [C1 vs. C2], 0.0034 [C2 vs. C3], Fig. S23a). The same trend was observed when weak positivity was counted as a positive marker (Fig. S23b). This result supports the idea that C2-AITL has atypical phenotypes and genotypes in TFH lymphomas. Although not statistically significant (adjusted *p* = 0.19, 0.22, and 0.07 for C1- vs. C2-AITL, C1-AITL vs. C3-AITL, and C2-AITL vs. C3-AITL, respectively, Fig. S24), the number of CD10-positive cases (12/26 [46.2%], 0/4 [0%], and 13/19 [68.4%] for C1, C2, and C3-AITL, respectively) suggests that the C3-subtype in the current analysis seemed to correspond to classical AITL. This finding was based on the frequently encountered CD10 positivity together with *IDH2* mutations, both of which have been previously reported as markers of classical AITL [40].

### Clinical features of genetic subtypes

**Fig. 6:**
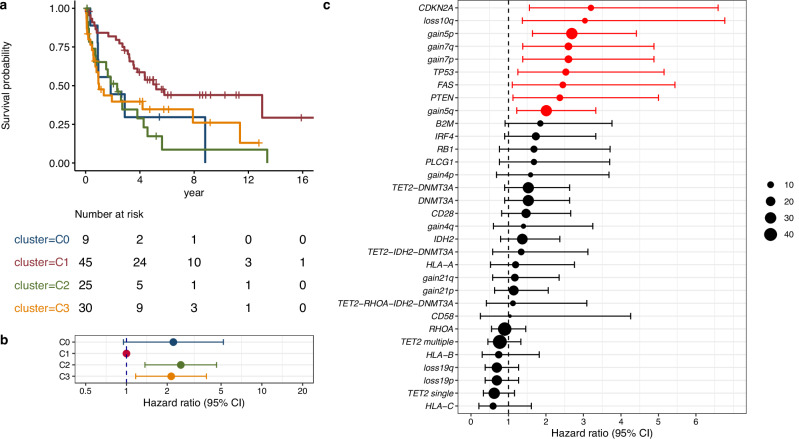
Survival outcomes across genetic subtypes. **a** Overall survival, stratified by genetic subtypes in all pathologies. **b** Forest plot of overall survival hazard ratio, stratified by genetic subtypes in all pathologies. **c** Forest plot for univariate Cox proportional hazards analysis for single or combination of genetic alterations.

We assessed the clinical features and outcomes of genetic subtypes. C2 (hazard ratio [HR], 2.52; 95% CI, 1.37–4.63) and C3 (HR, 2.14; 95% CI, 1.17–3.89) exhibited clinical inferior overall survival compared to C1 (Fig. 6a, b). Among AITL subgroups, C3 was a poor prognostic subtype when compared to C1 (HR, 2.7; 95% CI, 1.35–5.38) (Fig. S25). In cluster C2, all patients, excluding censored cases, died within 4 years, with a HR of 2.45 for C2 versus C1, although statistical significance was not reached due to the small sample size (7 cases). These results were similar when analyzed in the entire nTFHL population (Fig. S26), We also evaluated the prognostic impact of individual genetic alterations. In the univariate analysis, loss of *CDKN2A*, *FAS*, *PTEN*, and chr 10q and gain of chr 5, chr 7, and *TP53* mutations, which were either hallmark genetic alterations of C2 or C3, were poor prognostic factors (Fig. 6c). In patients with AITL, chr 5 gain was the only poor prognostic factor (Fig. S27). Furthermore, we explored the prognostic effect of chr 5 gain in another cohort and observed inferior progression-free survival compared to that in the cohort without chr 5 gain (*p* = 0.044, Fig. S28). Compared to C1- and C2- AITL, C3-AITL also had a higher frequency of rash and C-reactive protein levels, which were identified as prognostic marker [41] (Table 1).

**Table 1:** Patient characteristics of AITL stratified based on genetic clusters.

Characteristic	C1, *N* = 43^a^	C2, *N* = 8^a^	C3, *N* = 30^a^	*p* value^b^
Age, years	69 (60, 76)	72 (54, 76)	71 (64, 80)	0.5
Stage				0.068
1	1 (2.4%)	1 (14%)	0 (0%)	
2	2 (4.8%)	2 (29%)	1 (3.6%)	
3	19 (45%)	1 (14%)	9 (32%)	
4	20 (48%)	3 (43%)	18 (64%)	
PIT				0.2
G1	3 (7.5%)	0 (0%)	0 (0%)	
G2	9 (22%)	3 (43%)	4 (15%)	
G3	16 (40%)	1 (14%)	7 (26%)	
G4	12 (30%)	3 (43%)	16 (59%)	
Age > 60	28 (67%)	5 (62%)	24 (86%)	0.14
LDH > ULN	34 (83%)	6 (75%)	24 (86%)	0.7
PS > 1	13 (31%)	3 (43%)	16 (57%)	0.095
Bone marrow invasion	12 (30%)	2 (29%)	7 (26%)	>0.9
Eosinophil count, ×10^9^ /L	124 (0, 278)	167 (82, 328)	197 (0, 392)	0.8
plasmacytosis	3 (9.1%)	0 (0%)	1 (4.8%)	>0.9
Hemoglobin, g/dL	12.15 (10.95, 13.33)	12.70 (11.70, 12.80)	11.60 (10.50, 12.10)	0.2
Platelet count, ×10^9^ /L	18 (12, 24)	15 (14, 22)	15 (11, 25)	0.7
White blood cell count, ×10^9^ /L	7150 (4300, 10,625)	5200 (4100, 6200)	7900 (5400, 10,100)	0.2
CRP, mg/dl	0.82 (0.39, 2.95)	0.65 (0.18, 1.16)	3.29 (1.49, 7.34)	**0.002**
sIL-2R, U/ml	5032 (1905, 7178)	4435 (2700, 5421)	9196 (4160, 11,475)	0.077
Positive direct Coombs test	12 (71%)	NA	9 (69%)	>0.9
Rash	12 (39%)	0 (0%)	12 (63%)	**0.033**
Pleural effusoin or ascites	4 (12%)	0 (0%)	6 (29%)	0.3

*PIT* prognostic index for PTCL-U, *LDH* lactate dehydrogenase, *ULN* upper limit of normal range, *PS* performance status, *CRP* C-reactive protein, *sIL-2R* soluble interleukin 2 receptor.

^a^Median (interquartile range); *n* (%).

^b^Kruskal-Wallis rank sum test; Fisher’s exact test.

Statistically significant *p* < 0.05 values are in bold.

### TME signature

**Fig. 7:**
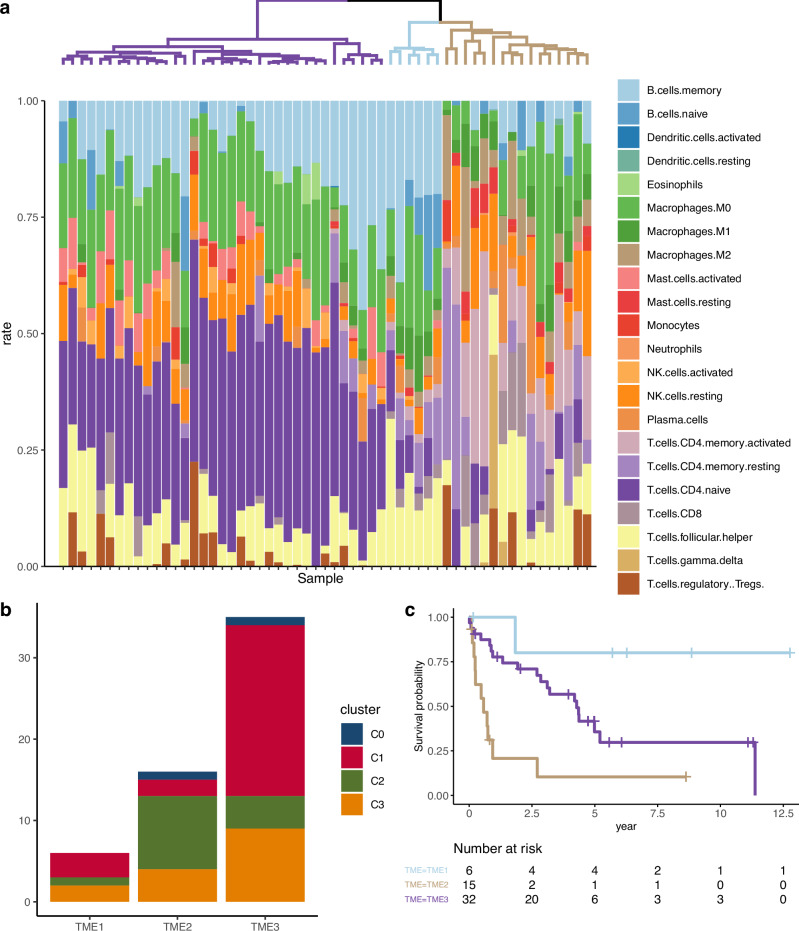
Tumor microenvironment signatures of PTCL. **a** Hierarchical clustering of tumor microenvironments. **b** Kaplan-Meyer curves of overall survival in each TME subtypes. **c** Genetic subtypes across TME subtypes.

We estimated the TME signature from the bulk RNA-seq data (*n* = 57) using the CIBERSORTx deconvolution method with the LM22 dataset [31]. The TME signatures of nTFHL and PTCL-NOS cells were divided into three groups (TMEs1-3) using hierarchical clustering. Increased number of naïve B cells, memory B cells, and TFH cells was a characteristic feature of the TME1 signature (Figs. 7a, S29, and S30), whereas the fraction of activated memory CD4^+^ T cells was significantly larger in the TME2 signature than in the other TME signatures (Fig. S29). The frequencies of activated memory CD4^+^ T cells also significantly correlated with those of CD8^+^ T cells, M1/M2 macrophages, resting mast cells, and neutrophils (Fig. S30). The fraction of CD4-naïve T cells and activated mast cells was larger in TME3 than in the other TMEs (Fig. S29). The frequencies of activated natural killer cells, eosinophils, and activated dendritic cells were significantly correlated with those of CD4-naïve T-cells (Fig. S30). To validate the cellular composition of the TME groups identified by CIBERSORTx, we performed IHC analysis for CD20 (B cells) and CD163 (M2 macrophages) and quantified the positive cells. The proportion of CD20-positive cells was highest in TME1, followed by TME3, and lowest in TME2 (31.1%, 2.3%, and 16.6% in TME1, TME2, and TME3, respectively; TME1 vs. TME2, *p* = 9.8 × 10^−^^5^; TME1 vs. TME3, *p* = 6.2 × 10^−^^3^; TME2 vs. TME3, *p* = 0.028; Fig. S31a). Conversely, CD163-positive cells were significantly more abundant in TME2 compared to TME1 and TME3 (8.6%, 23.9%, and 6.1% in TME1, TME2, and TME3, respectively; TME1 vs. TME2, *p* = 0.036; TME2 vs. TME3, *p* = 0.021; Fig. S31b). These results confirmed the increase in B cells in TME1 and M2 macrophages in TME2, supporting the validity of the TME classifications. Notably, the histological diagnosis was AITL in all TME1, 85.7% of TME3, and 31.3% of TME2 signatures (Fig. S32). We also evaluated the correlation between genomic subtypes and TME signatures using pairwise Fisher’s exact tests. C2 had significantly more TME2 signatures than C1 (64.3% vs. 7.7%), whereas C1 had significantly more TME3 signatures than C2 (80.8% vs. 28.6%, *p* = 0.006) (Fig. 7b). GSEA analysis between TME2-PTCL-NOS and TME3-PTCL-NOS revealed enrichment of several pathways responsible for macrophage-rich TME, as described previously, including IL-4/IL-13 and MTORC1 signaling (Fig. S33a, b) [42, 43]. Notably, the TME2 signature was more significantly associated with shorter survival than the TME3 signature (HR: 3.4, 95% CI 1.6–7.5; Fig. 7c, S34).

## Discussion

We performed an integrated multiomics analysis of nTFHL and PTCL-NOS specimens and identified several previously unrecognized links between genetic subtypes, TMEs, and clinical features. Our approach revealed that the genetically defined subgroup C3-AITL had distinct immunohistochemical features and clinical parameters, exhibiting inferior survival outcomes. Conversely, patients with C1-AITL had relatively better survival.

*IDH2* mutations are more frequent in C3-AITL than in C1-AITL, leading to accumulation of the oncometabolite 2-hydroxyglutarate, which suppresses TET activity and causes genomic instability and aneuploidy [44, 45]. Chr5 gain, being more frequent in C3-AITL than in C1-AITL, might also contribute to poor survival in C3 due to increased signaling of IL-4/IL-13, which is located on 5q31, via a direct gene dosage effect of aneuploidy [46]. This is supported by differentially expressed genes analysis of C3- and C1-AITL, which indicated increased M2 macrophage polarization via IL-4/IL-13-induced alternative activation pathway [47]. IL-4, in collaboration with IL-21, is also essential for germinal center B cells maturation [48]; we recently reported the importance of the interaction between germinal center B cells bearing *Tet2* mutations and tumor cells in the pathogenesis of AITL using a murine model [49]. Interestingly, phenotypic links between higher CD10 positivity and increased IL-4 secretion have also been observed in non-neoplastic TFH cells [50]. Higher levels of CRP in C3-AITL patients might be explained by the higher frequency of either *IDH2* or multiple *TET2* mutations, as Tet2 recruits Hdac2 and represses *IL-6* transcription via histone deacetylation [51]. Thus, the gene expression patterns, immunophenotypes, and clinical features of the C3-genetic subtype in the current study extend to previous findings on *IDH2*-mutated AITL [36, 40].

AITL with both *IDH2* and *TET2* mutations, corresponding to the C3 subtype, exhibits VEGF signaling pathway activation, leading to proliferation of HEVs compared to *TET2* single mutations or WT cases [15]. This observation explains the genomic and pathological differences between AITL and the other nTFHL subtypes. However, *IDH2* mutations are found in only 20–30% of AITL cases, and other AITL cases without *IDH2* mutations, mainly the C1 subtype, can also have increased angiogenesis. Here, we demonstrated that C1 was significantly associated with TME3, which is characterized by an increased proportion of mast cells and naïve T cells. Angiogenesis in this subgroup may be due to an increased proportion of mast cells, which promote angiogenesis by releasing angiogenic mediators in solid tumors [52]. Given the abundance of HEV and non-tumor T-cells in AITL [53], a higher proportion of naïve T-cells within the C1-TME is unsurprising, as naïve T-cells enter lymph nodes via the bloodstream through HEV [54].

*TET2* mutations are established signatures of nTFHL, particularly AITL, and have been extensively investigated for profiling. Our results revealed that a subset of patients with a high VAF ratio of *TET2* to *RHOA* mutations, possibly indicating the expansion of non-tumor cells carrying *TET2*-CH, had a significantly worse prognosis than the low-ratio group. Notably, hypomethylating agents (HMAs) may be able to target not only tumor cells but also *TET2*-CH-derived non-tumor cells [55]. Several clinical trials have reported the efficacy of HMAs, including oral azacitidine and guadecitabine [33, 56]. Indeed, *TET2* mutations have been associated with the efficacy of oral azacitidine in AITL [56].

Here, we validated the previously defined high-risk subset of PTCL-NOS with aneuploidy and tumor suppressor gene alterations as C2 subtype. Furthermore, we clarified the previously unrecognized overlap between this entity and PTCL-NOS with extra copies of *IRF4* corresponding to increased *MYC* expression, known in another context as a poor prognostic subtype [35, 57].

We also offer new insights into the possible overlap of two high-risk groups, C2 and TME2, because TME2 in the current study was equivalent to non-B/non-DC or macrophage-associated TME in previous studies [43, 58]. Previous studies [26, 36] have shown that PTCL-GATA3 is often characterized by genomic abnormalities corresponding to the C2 subtype. GATA3 is a critical transcription factor in TH2 cells and contributes to the production of cytokines that induce M2 macrophages [47], such as IL-4 and IL-13, which may explain the observed correlation between the C2 subtype and TME2 in this study. Increased mTORC1 signaling resulting from dysregulation of PI3K/AKT mTOR signaling in the C2 genetic subtype is another possible explanation for the macrophage-rich TME2 [42].

In conclusion, our integrative approach successfully identified the molecular subtypes and distinct TME signatures associated with specific clinical characteristics, tumor cell immunophenotypes, and prognostic implications in PTCL. This comprehensive framework lays a foundation for advancing precise treatment strategies for PTCL, potentially leading to improved patient outcomes.

## Supplementary information

Supplementary Methods and Results

Table S1

Table S2

Table S3

Table S4

Table S5

Table S6

## Data Availability

The WES data are deposited in the European Genome-Phenome Archive and are available upon request.
