# Supplementary material for: Discrete genetic subtypes and tumor microenvironment signatures correlate with peripheral T-cell lymphoma outcomes
Source: Leukemia. 2025 Mar 31;39(5):1184–95. doi: 10.1038/s41375-025-02563-0 (PMC12055585; doi:10.1038/s41375-025-02563-0)
Supplement: Supplementary file 1 — Supplementary Methods and Results [file 41375_2025_2563_MOESM1_ESM.docx]

**Supplementary methods**

**Overview of the strategy to optimize somatic mutation detection in low-tumor-content samples**

Our strategy to address low tumor content and minimize the risk of false positives consists of four steps: (1) applying filters based on sequencing quality (coverage, base call and mapping quality, strand ratio), (2) using Empirical Bayesian Mutation Calling (EBcall) (<https://github.com/friend1ws/EBCall>)^1^, which employs an empirical Bayesian framework using multiple non-paired normal samples to effectively distinguish true somatic mutations from sequencing errors, hereby reducing false positives, (3) somatic mutation prediction using a random forest model validated on a separate dataset. The performance of the model was 98.3% specificity and 86.7% sensitivity (Figure S35). This indicates a trend toward minimizing false positives, potentially at the expense of increasing false negatives. Furthermore, feature importance analysis revealed that tumor VAF plays a major role (Figure S36), sometimes causing high-VAF somatic mutations to be misclassified as germline and leading to false negatives. (4) To address these false negatives, we established specific “rescue” criteria for reclassifying germline-misclassified variants as somatic. By combining EBcall for reducing false positives derived from sequencing errors, a random forest-based model for somatic mutation prediction, and a targeted rescue step for misclassified germline variants, our approach effectively minimizes false positives and ensures accurate somatic mutation detection even in the context of low tumor content.

**Variant calling of single nucleotide variants and short indels**

Mapping and variant calling were conducted using the Genomon pipeline (<https://genomon-project.github.io/GenomonPagesR/>). The reference sequence used was hg19. For the 18 samples with paired normal controls, paired analysis was performed. Four of the 18 tumor samples with paired normal controls, used for somatic variant prediction, underwent whole-genome amplification using REPLI-g Kit (Qiagen) and were excluded from subsequent copy number analysis owing to their unsuitability. For 115 samples without paired normal controls, a tumor-only analysis was performed. The criteria for a tumor-only analysis were as follows: (i) depth (coverage) ≥ 8, (ii) base quality ≥ 15, (iii) variant read count ≥ 4, (iv) variant allele frequencies (VAFs) > 2%, (v) mapping quality ≥ 20, (vi) reads were excluded with Samtool flags indicating unmapped secondary mapping, QC failure, and duplicates. The paired sample analysis incorporated the following criteria into the tumor-only analysis: (i) Variant allele frequency < 0.1 in the matched normal control, (ii) Fisher’s exact test P-value < 0.1 for the distribution of alternate and reference alleles in tumor and matched normal samples. Following variant calling, we filtered based on the following two conditions: (i) 0 < strand ratio < 1, (ii) Ebcall P value < 10^-4^. We used 36 non-tumor samples for EBCall. Visual inspection of candidate mutations was performed using IGV (v.2.4.0), and errors were manually excluded.

**Developing the somatic mutation prediction model**

We built a somatic mutation predictor using a random forest model (R caret package v.6.0.84) to discriminate somatic SNVs and short indels from the germline in unpaired samples. Among the 18 samples with paired normal controls, we conducted mutation analysis both in the paired analysis and tumor-only analysis without paired normal controls. Mutations identified in both paired and tumor-only analyses were labeled as somatic mutations, whereas those identified in tumor-only but not in paired analysis were labeled as germline mutations. Mutations identified in 15 samples were randomly split into a training dataset (75%) and a test dataset (25%). Only the training dataset was used to construct a model for predicting somatic mutations. Mutations identified in the remaining three samples were reserved for performance evaluation. We constructed a model using the following 31 variables to predict somatic and germline mutations: Allele frequencies of variants in the National Heart, Lung, and Blood Institute Exome Sequencing Project (esp6500siv2_all), those in the 1000 Genomes Project (1000g2010nov_all, 1000g2014oct_all, 1000g2014oct_afr, 1000g2014oct_eas, and 1000g2014oct_eur), those in the Tohoku Medical Megabank Organization (ToMMo-4. 7kjpn)^2,3^, and those in the Human Genetic Variation Database (HGVD_2013, HGVD_2016). Whether the variants are curated in the database or not (snp131 and snp138 from Single Nucleotide Polymorphism, and cosmic68wgs, cosmic70 from Database and Catalogue Of Somatic Mutations In Cancer). Scores from in silico pathogenicity prediction tools (SIFT, Polyphen2 HDIV, Polyphen2 HVAR, LRT, MutationTaster, MutationAssessor, FATHMM, RadialSVM, LR, VEST3, CADD raw, GERP++ RS, phyloP46way placental, phyloP100way vertebrate, SiPhy 29way logOdds). Read depth, variant allele frequencies and p-values of Ebcall in each variant. Imputation of variables with missing values for each mutation was performed using the R MICE package v.3.13.0. To evaluate model performance, we used the test dataset (25%) that was not used for model construction (Test 1), and mutations derived from the three samples for independent model evaluation (Test 2) (Figure S35). Variables with high feature importance in the prediction model were VAFs in tumors, MAFs in ToMMO-4.7kJPN, and EB call P value. (Figure S36)

**Reclassification of mutations initially predicted as germline to rescued somatic mutations**

After somatic variant prediction, to mitigate the misclassification of high VAF cases as germline, we rescued variants that met at least one of the following criteria: (a) known hotspot mutations in driver genes, (b) truncating mutations (nonsense or frameshift indels) in tumor suppressor genes, or (c) mutations in recurrent driver genes with a minor allele frequency of < 0.002 across all databases, including esp6500siv2_all, 1000g2010nov_all, 1000g2014oct_all, 1000g2014oct_afr, 1000g2014oct_eas, 1000g2014oct_eur, HGVD_2013, HGVD_2016, and ToMMo-4. 7kjpn.

**Results of somatic mutation prediction and characteristics of rescued somatic mutations**

Of the 4,764 candidate mutations identified by the model, 102 were reclassified as somatic based on manual inspection (hereafter referred to as “rescued somatic mutations”) (Figure S37). These rescued mutations had significantly higher VAFs than model-predicted somatic mutations (Figure S38, 36% vs. 11.7%, p = 9.57 × 10⁻¹²³) but lower VAFs than germline mutations (36% vs. 51.4%, p = 1.49 × 10⁻⁷⁶), and their minor allele frequencies in ToMMo-4. 7kjpn were significantly lower than germline mutations (Figure S39, median 0.0785 vs. 0, p = 2.63 × 10⁻¹⁵). *TET2* mutations constituted 44.1% of rescued mutations, followed by *DNMT3A* and *TP53* (Figure S40). The high VAFs observed in rescued mutations were presumed to reflect clonal hematopoiesis in non-tumor cells for *TET2* and *DNMT3A* mutations, and concomitant *TP53* loss in the contralateral allele for *TP53* mutations.

**Varinat calling of structural variants**

Structural variant calling was conducted using GenomonSV (<https://github.com/Genomon-Project/GenomonSV>) within the Genomon pipeline (version 2.6.2). Variants were identified based on the following criteria: (1) a minimum junction count of ≥2, (2) removal of candidate variants detected in a panel of 36 normal samples with junction reads ≥2, (3) a minimum overhang of ≥30 bp between breakpoints, (4) a variant allele frequency (VAF) ≥0.07, and (5) an inversion size >1000 bp. Mapping errors were manually reviewed and excluded using the IGV.

**Copy number analysis using whole exome sequencing data**

We detected substantial copy number alterations (CNAs) using GISTIC 2.0^4^ from segmental copy number model data, which were built using CNV GATK4 tools v.4.1.3 (<http://github.com/gatk-workflows/gatk4-somatic-cnvs>) from the bam files. Annotation intervals were created based on the bed files of the capture baits (Sureselect V7) with 100 base-pair padding. Read counts were collected from normal control samples to create a panel of normals. Read counts were collected from tumor samples' BAM files and adjusted with the panel of normals to obtain denoised copy ratios. Allelic counts were collected from tumor BAM files, and model segments were derived from these and the denoised copy ratios. GISTIC 2.0 was executed with the following parameters: -ta 0.137 -td 0.152 -broad 1 -rx 1 -armpeel 0 -js 15 -scent none. The threshold cutoff value was derived by estimating one-copy gain or loss at 20% tumor purity. To detect focal CNAs, we initially excluded FFPE samples to avoid calling false CNA events. This was because the number of segmentations in the copy number estimation model was significantly higher in FFPE samples than in FF samples (Figure S41). We detected twenty-five focal copy number changes that were significant changes using this method (Figure S8). Subsequently, we examined the presence of significant focal CNAs in FFPE samples and found that the frequencies of focal CNAs did not significantly differ across sample types (Figure S42.).

**Criteria for candidate driver genes**

Genes that met one of the following conditions were designated as candidate driver genes; mutations in other genes were classified as passenger mutations. (i) FDR q value < 0.1 in MutSig2CV, (ii) FDR q value < 0.1 in GISTIC2^4^, (iii) mutations observed in two or more samples have previously been identified as recurrent driver genes in lymphoma genomic research, (iv) not meeting (i), (ii), or (iii) but were previously identified as recurrent driver genes in previous genomic research related to PTCL, (v) members of a gene family, one of which was identified as a candidate driver gene meeting criterion (i), (ii), (iii), or (iv).

**Exclusivity and co-occurrence of driver genetic alterations**

For each genetic alteration, the significance of co-occurrence or mutual exclusivity with other genes and arm-level CNAs was evaluated using Fisher’s exact test, with multiple testing correction performed using the Benjamini-Hochberg method.

**RNA sequencing**

RNA was extracted from FFPE specimens using the RNAstorm FFPE kit (Cell Data Sciences). RNA quality was assessed using TapeStation (Agilent). Samples in which the fraction of RNA longer than 200 nucleotides was greater than 35% were used for library preparation (n = 57). Libraries were prepared using an Illumina RNA Prep with Enrichment (L) Tagmentation kit (Illumina). Adapter trimming was performed using Trinity (v.2.13.2)/Trimmomatic and quality was evaluated using FastQC v.0.11.9 (<http://www.bioinformatics.babraham.ac.uk/projects/fastqc>). Reads were mapped to hg19 using STAR (v.2.6.1). Ribosomal/transfer RNA reads were removed using Bedtools (v.2.27.1). Read counts were obtained using the FeatureCount software (Subread v.2.0.3). TMM normalization was performed using edgeR (v.3.28.1).

**References**

1 Shiraishi Y, Sato Y, Chiba K, Okuno Y, Nagata Y, Yoshida K *et al.* An empirical Bayesian framework for somatic mutation detection from cancer genome sequencing data. *Nucleic Acids Res* 2013; **41**. doi:10.1093/NAR/GKT126.

2 Kuriyama S, Yaegashi N, Nagami F, Arai T, Kawaguchi Y, Osumi N *et al.* The Tohoku Medical Megabank Project: Design and Mission. *J Epidemiol* 2016; **26**: 493–511.

3 Tadaka S, Katsuoka F, Ueki M, Kojima K, Makino S, Saito S *et al.* 3.5KJPNv2: an allele frequency panel of 3552 Japanese individuals including the X chromosome. *Hum Genome Var* 2019; **6**: 28.

4 Mermel CH, Schumacher SE, Hill B, Meyerson ML, Beroukhim R, Getz G. GISTIC2.0 facilitates sensitive and confident localization of the targets of focal somatic copy-number alteration in human cancers. *Genome Biol* 2011; **12**: R41.

**Supplementary figures**

**Figure S1. Sample information.**

(a) Samples used for whole exome sequencing.

(b) Independent cohort data set to test the prognostic impact of arm-level CNAs.

AITL, angioimmunoblastic T-cell lymphoma; FTCL, follicular T-cell lymphoma; nPTCL with TFH phenotype, nodal peripheral T-cell lymphoma with T follicular helper phenotype; nTFHL unclassifiable, nodal T-follicular helper lymphoma, unclassifiable; PTCL-NOS, peripheral T-cell lymphoma not otherwise specified.


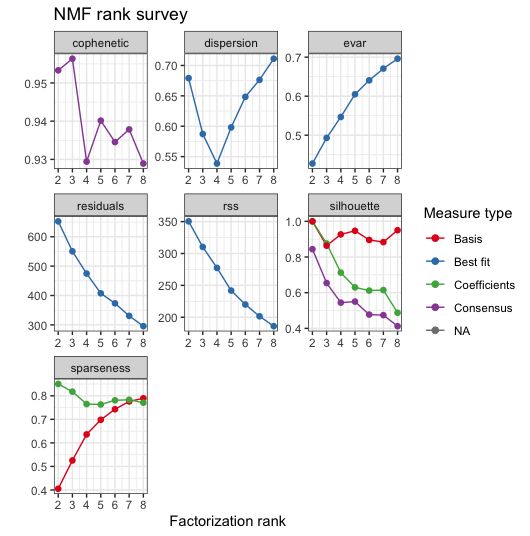


**Figure S2. Non-negative matrix factorization rank survey plot of quality measures.**

The X-axis indicates the number of clusters. NMF, Non-negative matrix factorization.

The number of clusters was determined to be three according to the cophenetic coefficient.

**Figure S3. Sequencing depth of whole exome sequencing.**

1. Average sequencing depth across samples. Red line indicates median of mean sequencing depth.
2. Percentage of target regions per case covered at the sequencing depth of ≥2, 10, 20, 39, 40, 50, and 100.

**Figure S4. Tumor mutational burden between fresh frozen and formalin-fixed paraffin-embedded specimens.** FF, freshly frozen; FFPE, formalin-fixed paraffin-embedded; ns, not significant.


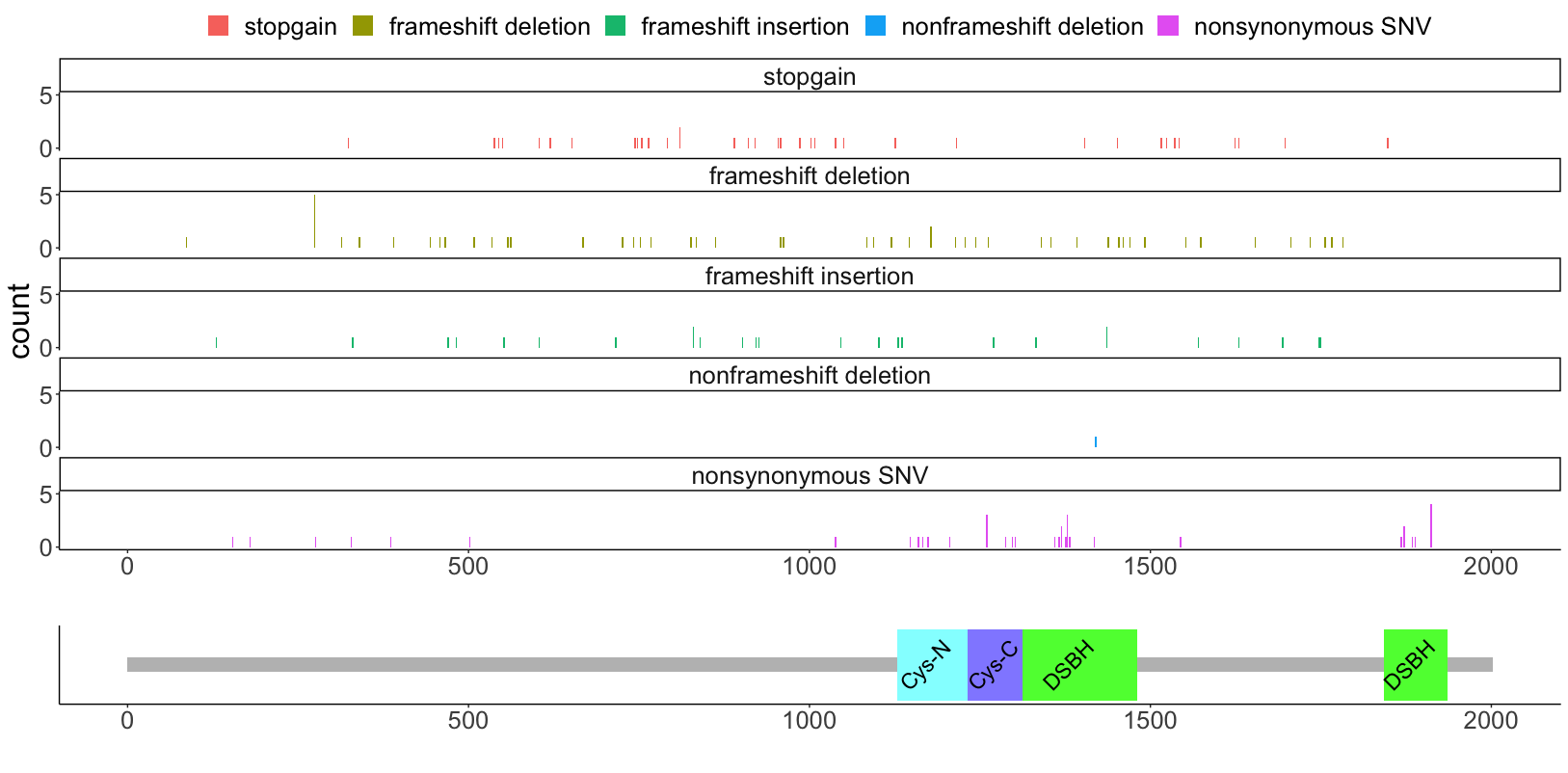


**Figure S5. Distribution of *TET2* mutations at protein levels.** Missense mutations accumulate in zinc-chelating residues, which are essential for TET2 structural formation, DNA recognition, and catalysis (Hu, *Cell* 2013; **155**: 1545–1555.) The x-axis denotes number of amino acids in the TET2 protein. SNV: single-nucleotide variants.


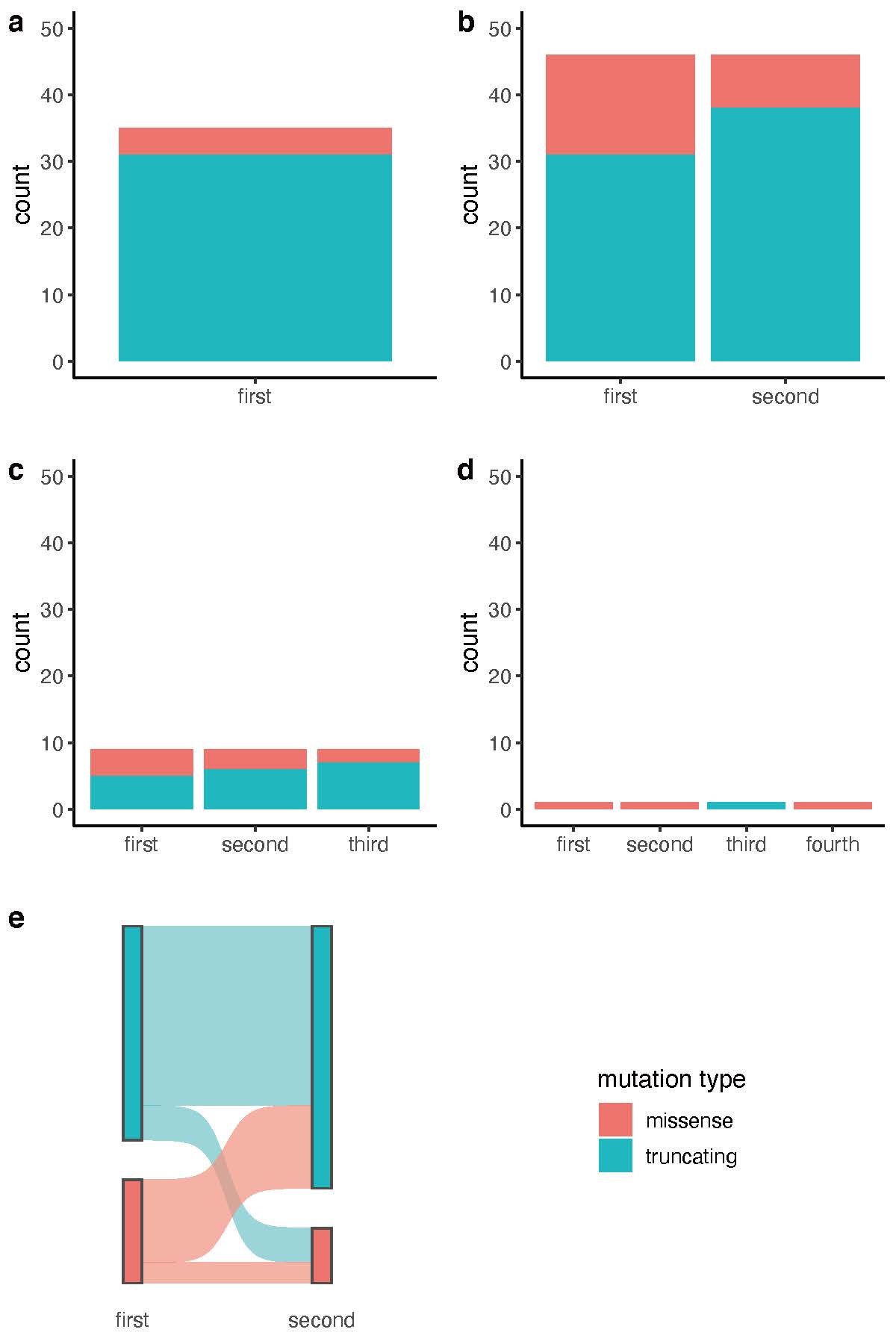


**Figure S6. Types of *TET2* mutations and acquired order.** The x-axis shows the acquired order estimated using variant allele frequencies. The y-axis shows the count of mutations. Data of patients with single, double, triple, and quadruple *TET2* mutations are shown in (a), (b), (c), and (d). (e) The Sankey plot shows a combination of first- and second-acquired mutation types.


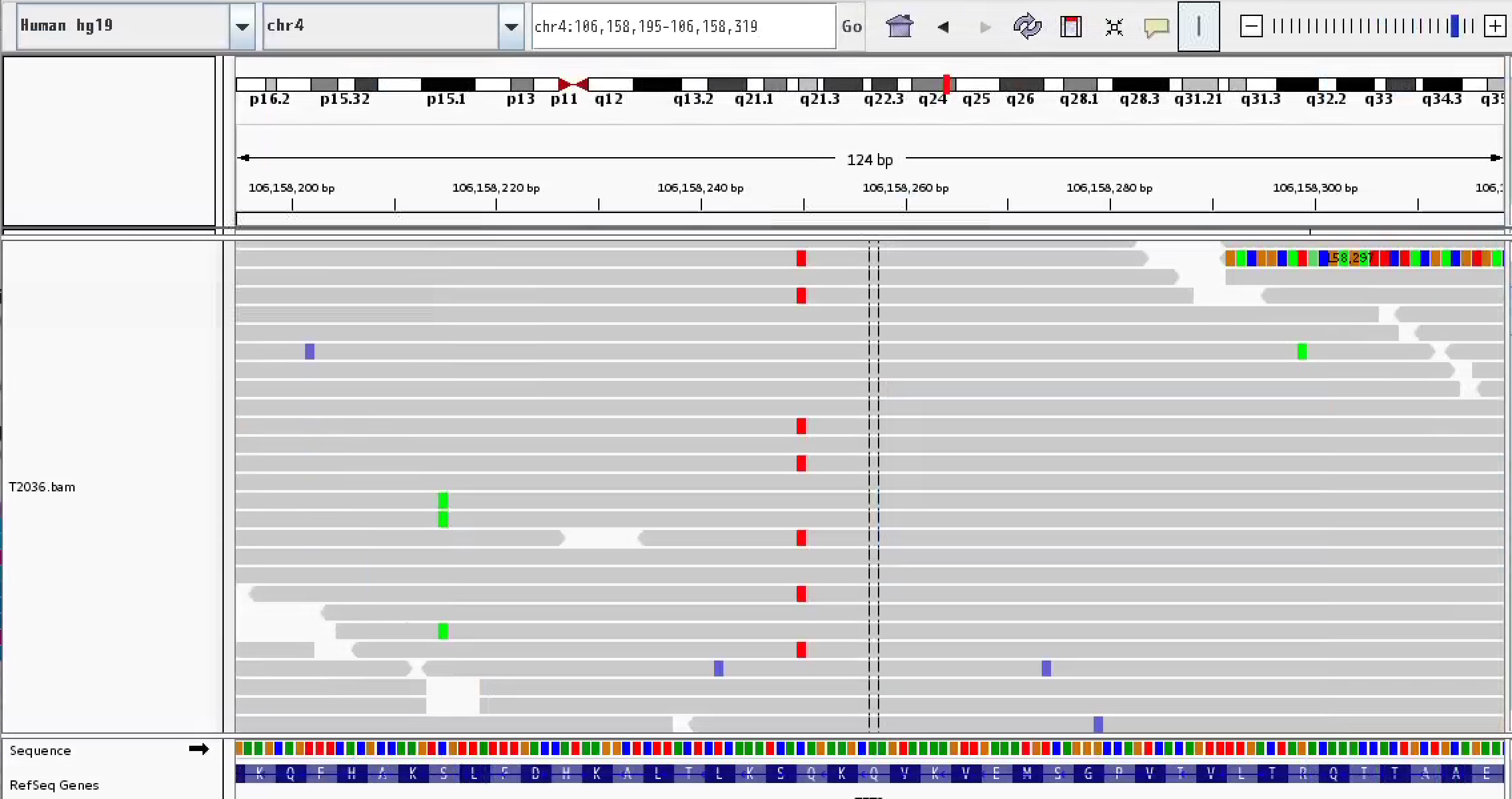


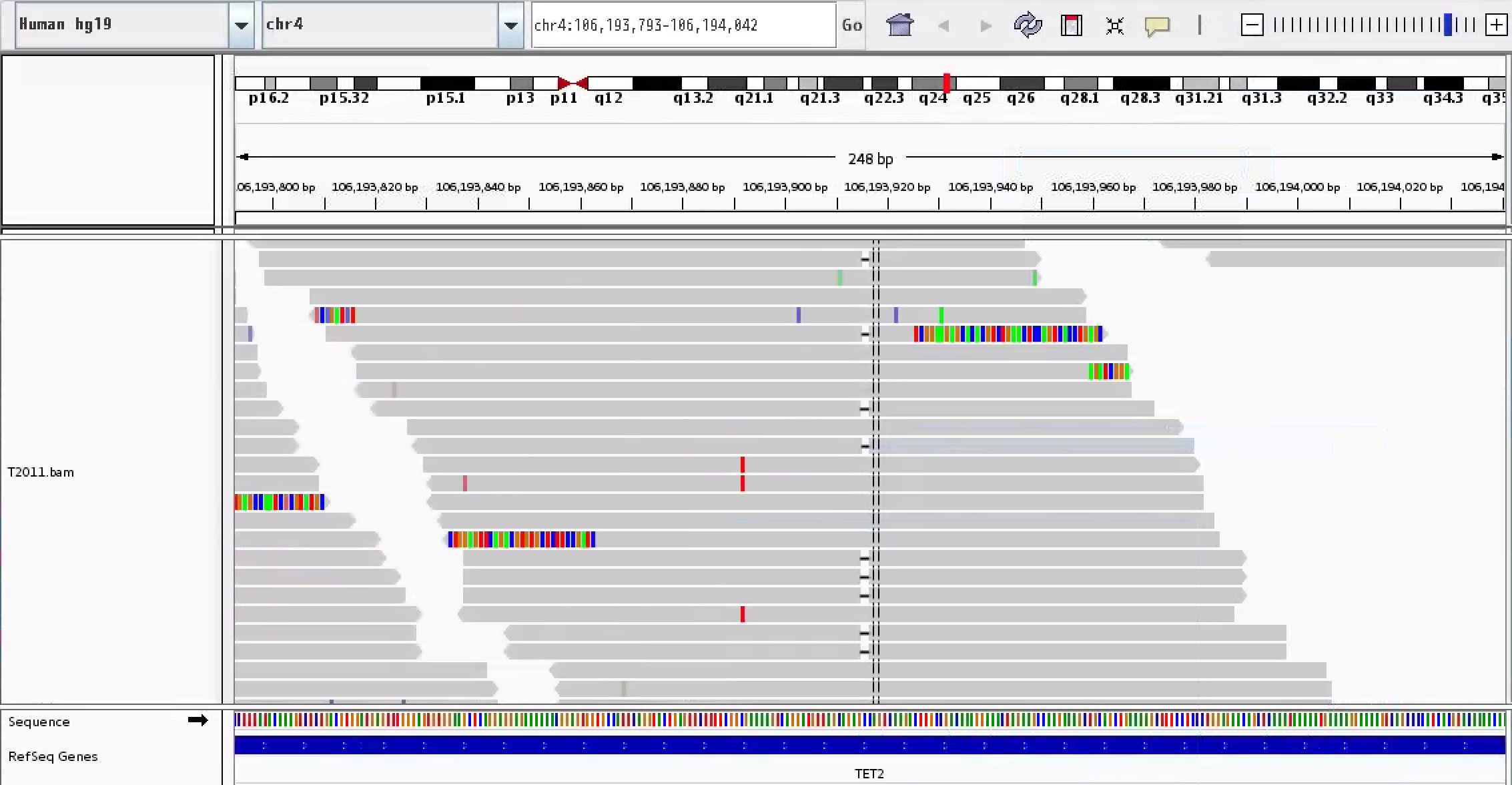


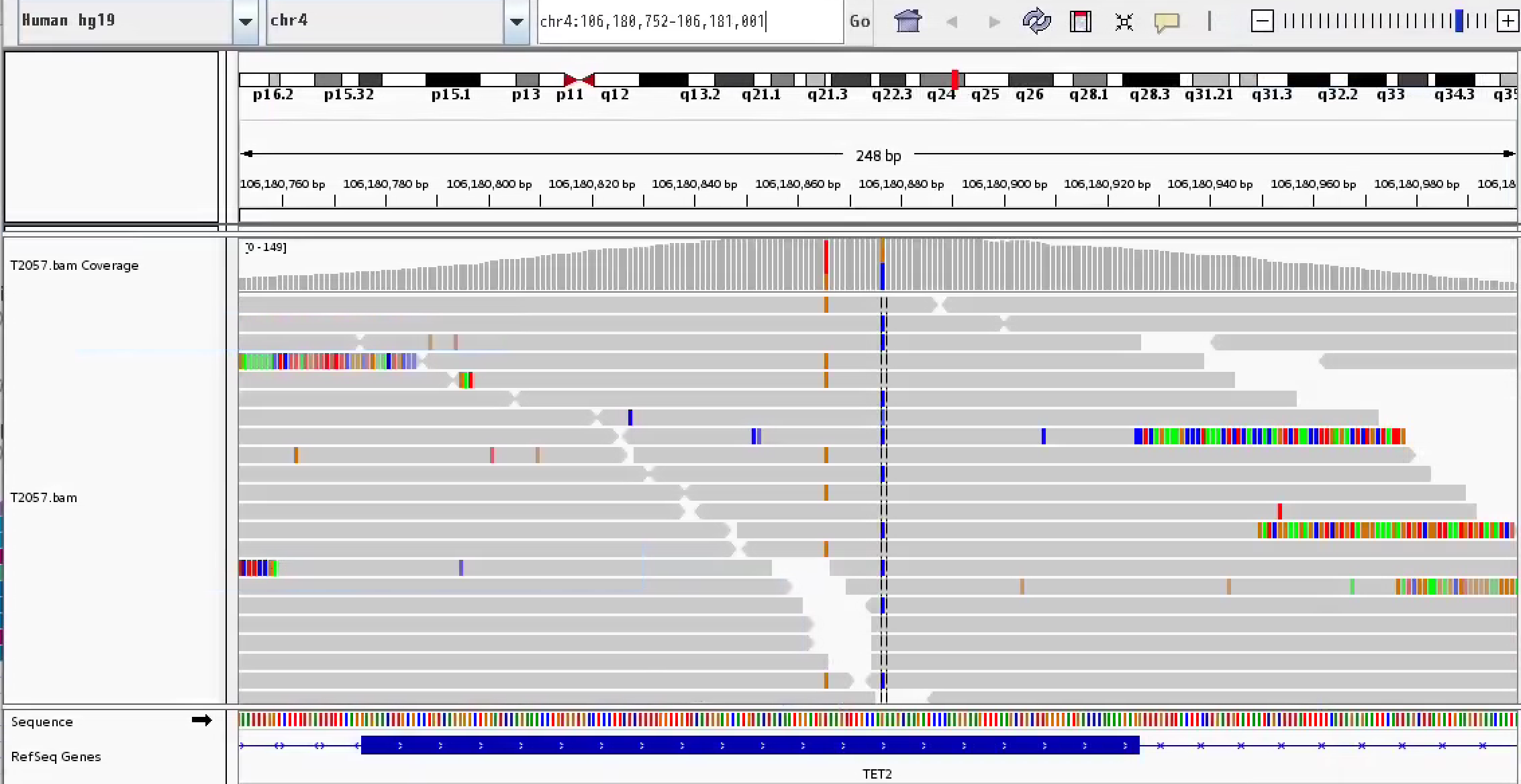


**Figure S7. Integrative Genome Viewer snapshot of double *TET2* mutations.** Among the pairs of multiple *TET2* mutations, cis/trans was evaluable in three pairs because the distances between *TET2* mutations in each pair were within the library read length (150 bp). All three pairs had different alleles that were interpreted as biallelic mutations in single clones or were derived from two different clones.

**Figure S8. Focal copy number alterations detected by GISTIC2.** The dotted line shows the threshold of significance (-log 10 q value = 1).

**Figure S9. mRNA expression levels of *CDKN2A* across genetic status.** Hetero, heterozygous loss; homo, homozygous loss; WT, wild type; CPM, counts per million; *, adjusted p-value < 0.05.


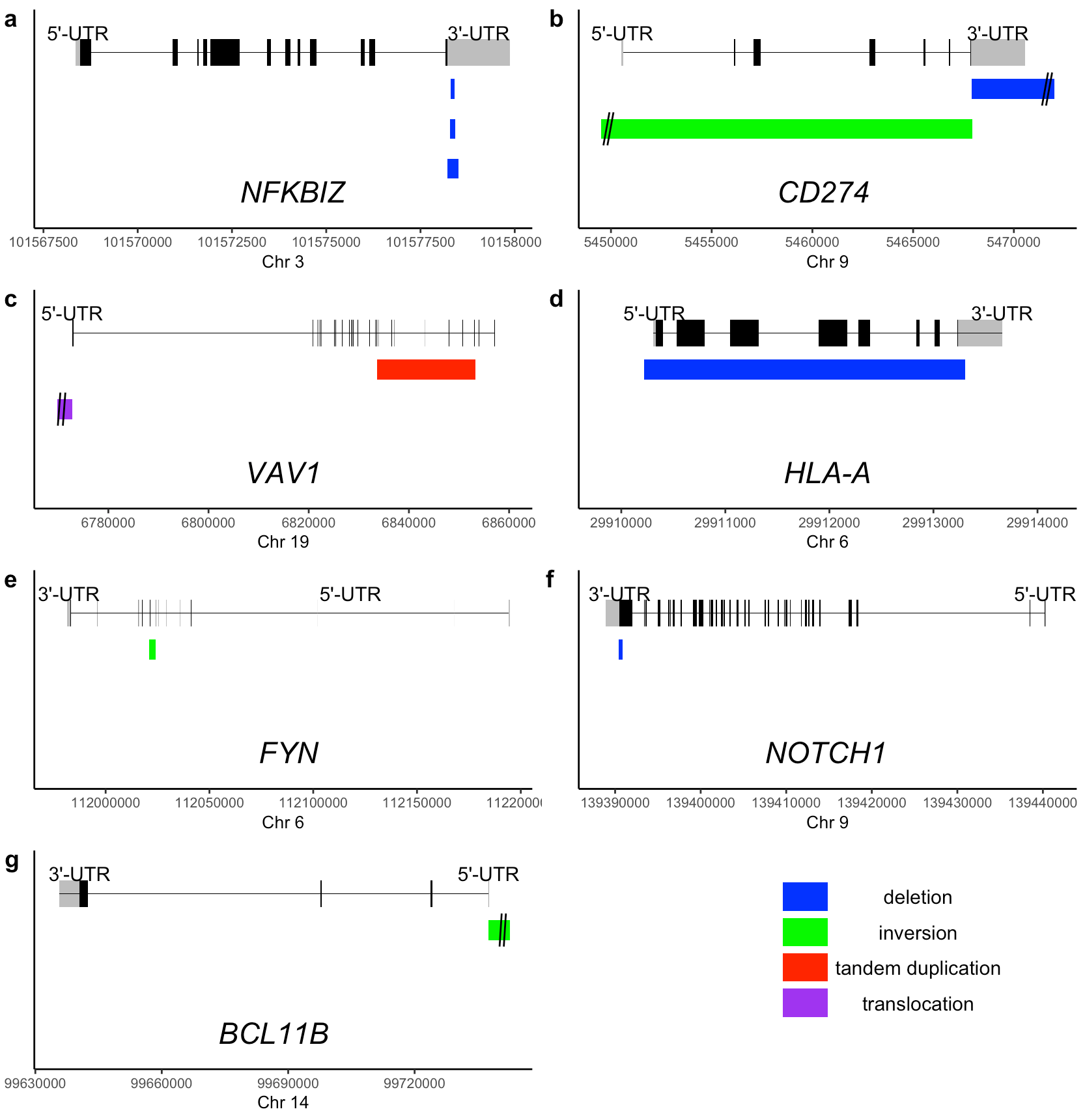


**Figure S10. Structural variants.**

UTR; untranslated region; Chr, chromosome.

**Figure S11. Frequencies of pathway alterations across PTCL subtypes.** AITL, angioimmunoblastic T-cell lymphoma; nTFHL, nodal peripheral T-cell lymphoma; PTCL-NOS, peripheral T-cell lymphoma not otherwise specified.

Adjusted p-value, *, **, ***, **** for < 0.05, 0.01, 0.001, 0.0001

**Figure S12. Number of driver gene alterations across genetic subtypes.**

* represents adjusted p-value < 0.05.

**Figure S13. Immunophenotypes, genetic clusters, and gene expression profiling of 35 PTCL-NOS cases.**

(a) immunophenotypes, (b) pathological diagnosis, (c) genetic subtypes, (d) gene set enrichment score of TFH, TBX-21, and GATA3 gene sets.

**Figure S14. Tumor mutational burden across the genetic subtypes.**

Adjusted p-value, *, *** for < 0.05, 0.001, respectively

**Figure S15.** **N**umber of arm-level copy number alterations across genetic subtypes.

Adjusted p-value, *, **** for < 0.05, 0.0001, respectively; CNAs, copy number alterations.


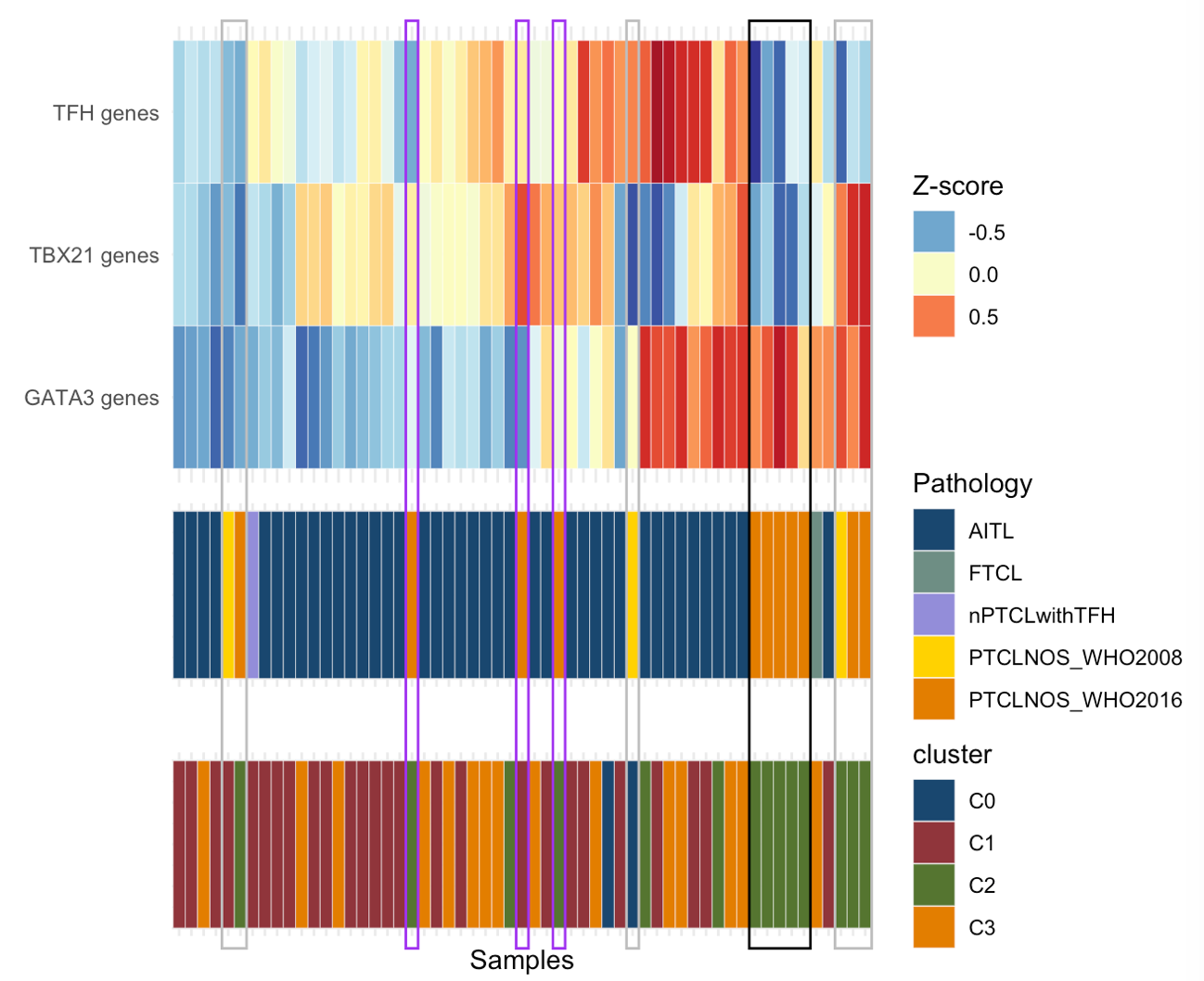


**Figure S16. Heatmap of gene set enrichment scores for TFH, TBX21-PTCL, and GATA3- PTCL gene sets**

Samples highlighted with black boxes represent GATA3-PTCL, purple boxes indicate TBX21-PTCL, and gray boxes denote PTCL cases that could not be classified based on GSVA analysis.


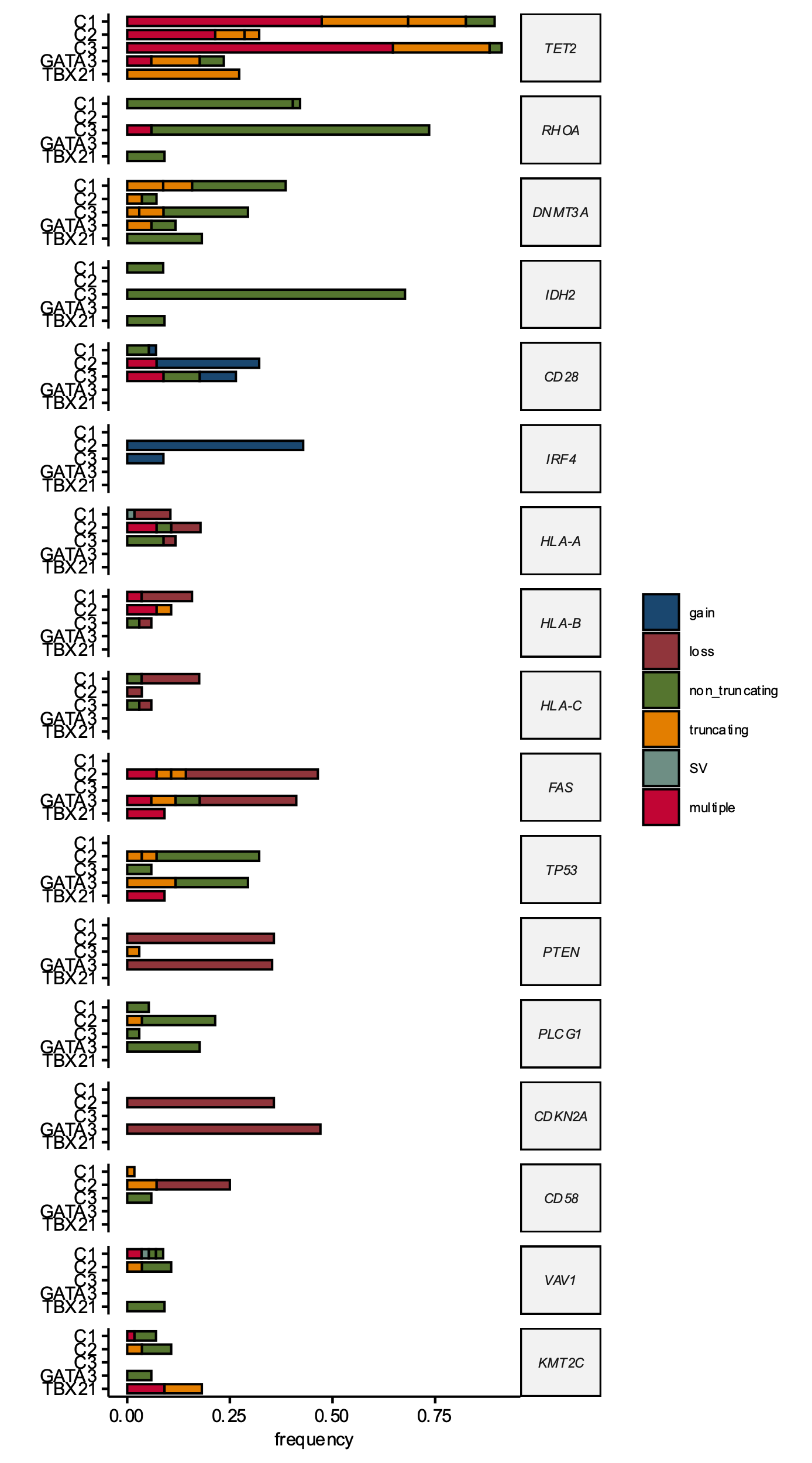


**Figure S17. Difference of single gene level alterations across genetic subtypes in this study and GATA3-/TBX21-PTCL subtypes.**

Comparison of genetic subtypes (C1–C3) identified in this study with previously reported PTCL molecular subtypes (GATA3 and TBX21) from Heavican et al. (Blood, 2019). Gene-level alterations in C1–C3 were derived from our dataset, while data for GATA3 and TBX21 subtypes were extracted from Figure 6 of Heavican’s paper.


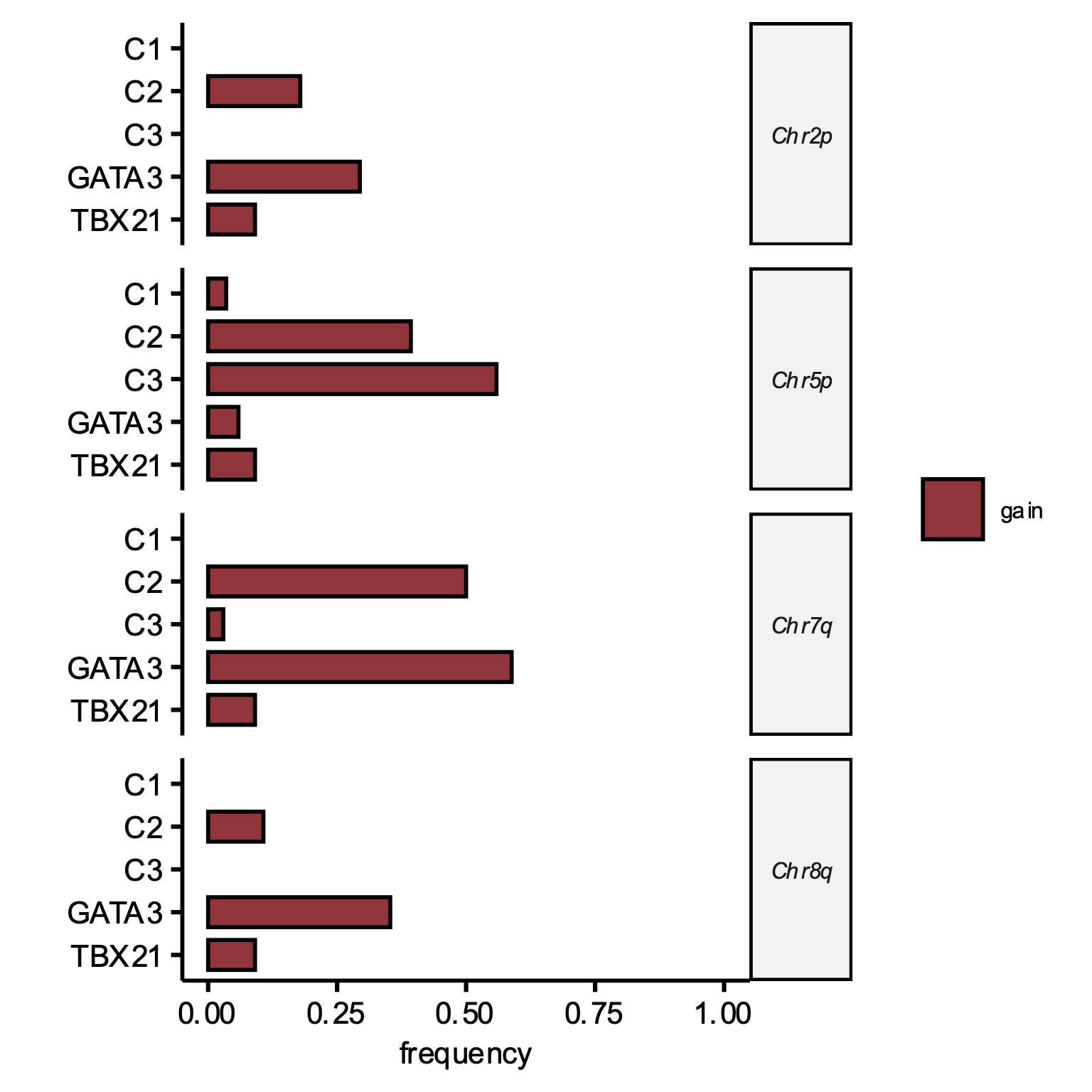


**Figure S18. Difference of chromosomal aberrations across genetic subtypes in this study and GATA3-/TBX21-PTCL subtypes.**

Arm-level copy number alterations (CNAs) in C1–C3 were derived from our dataset, while data for GATA3 and TBX21 subtypes were extracted from Figure 6 of Heavican et al. (Blood, 2019).

**Figure S19. The immunohistochemical analysis of 6 TFH markers across genetic subtypes in nTFHLs.**

**Figure S20. mRNA expression levels of *MYC* stratified by IRF4 status.**

CPM, counts per million; * denotes p-value <0.05

**Figure S21. Result of Metascape analysis using differentially expressed genes between C3- and C1-AITL.**

**Figure S22. Six TFH markers in AITL, nTFHL other than AITL, and CD4-positive PTCL-NOS**.


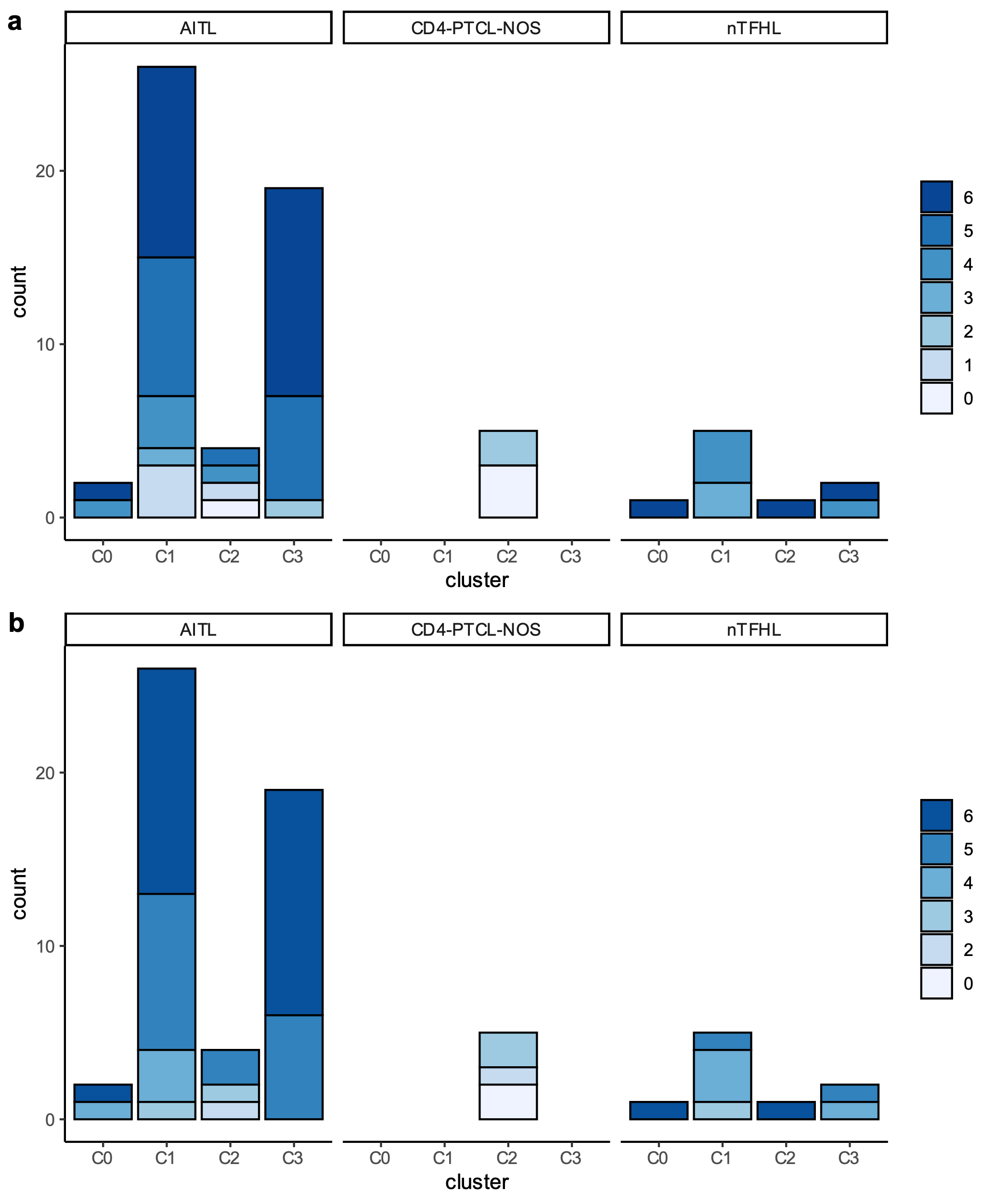


**Figure S23. Number of positive TFH markers across genetic subtypes and pathological diagnoses.**

Immunohistochemical characteristics of genetic subtypes were analyzed in patients with at least 5 of 6 evaluable TFH markers. (a) Only positive markers were counted. (b) Weak positive markers were also counted.


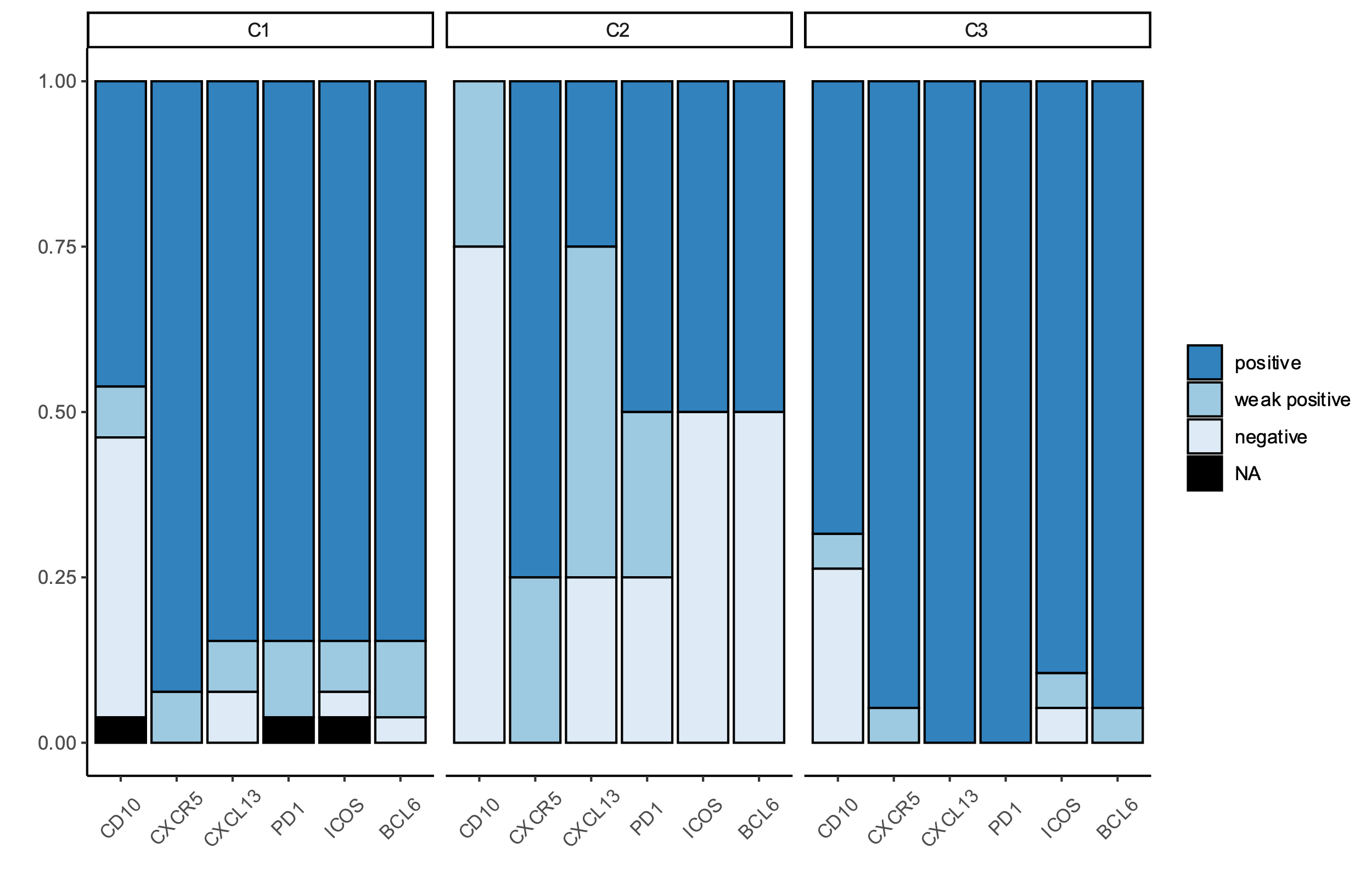


**Figure S24. Six TFH markers by genetic subtype of AITL.**

Immunohistochemical characteristics of genetic subtypes were analyzed in patients with at least 5 of 6 evaluable TFH markers. C0-AITL cases meeting this criterion were limited to two cases and were therefore excluded from the analysis.

(a)

(b)

**Figure S25. Overall survival, stratified by genetic subtypes in AITL.**

1. Kaplan-Meier curve, (b) **Forest plot of overall survival hazard ratios**

(a)

**
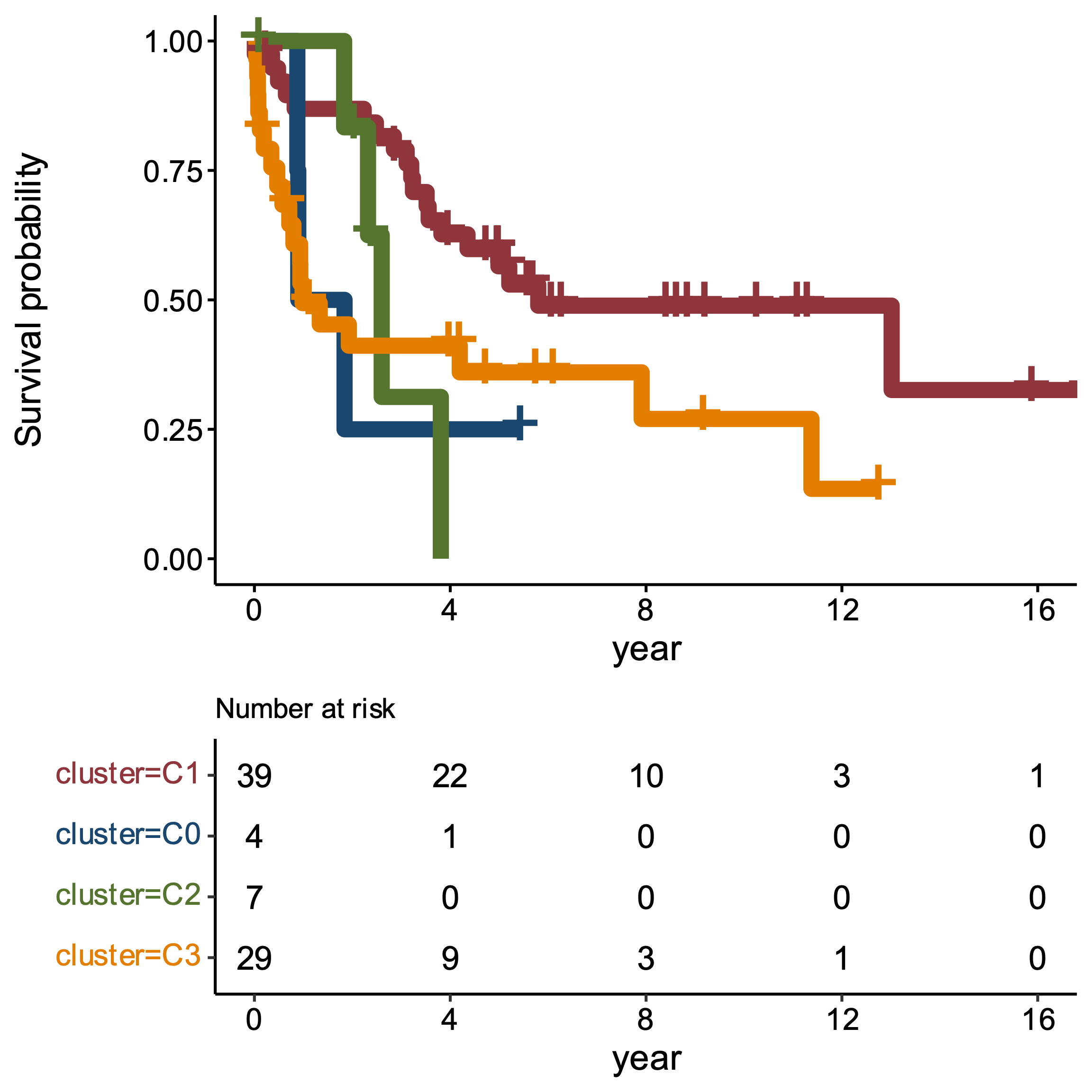
**

(b)

**
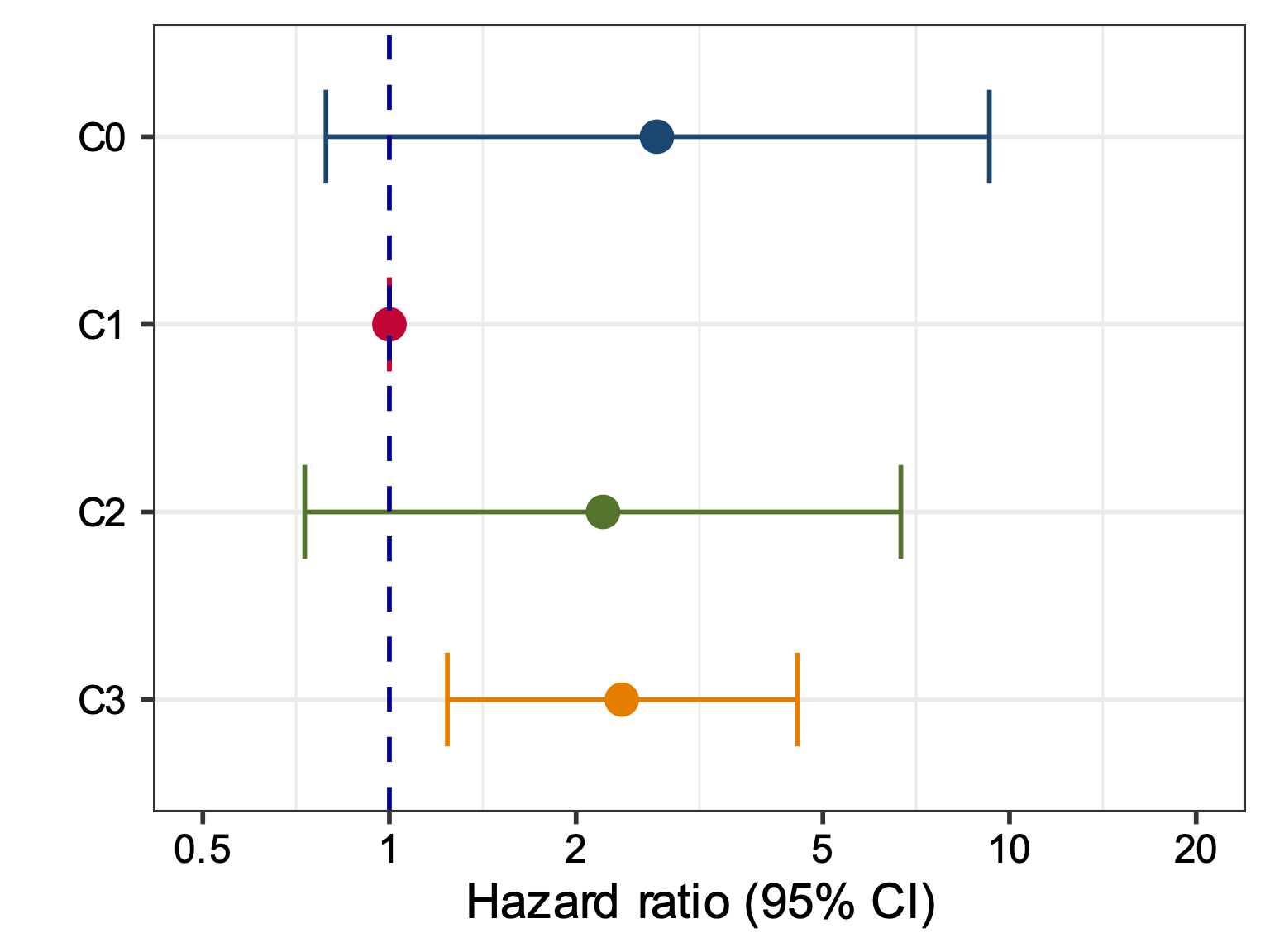
**

**Figure S26. Overall survival, stratified by genetic subtypes in nTFHL.**

(a)Kaplan-Meier curve, (b) **Forest plot of overall survival hazard ratios**

**Figure S27. Forest plot for univariate Cox proportional hazards analysis for single or combination of genetic alterations in patients with AITL.**

Dots of significantly different genes are indicated in red (adjusted p-values). The size of the dots indicates the number of patients with mutations for each genomic alteration.

**Figure S28. Survival curve stratified by gain of chromosome 5 in a validation cohort.**

1. Overall survival; (b) progression-free survival. A total of 48 patients with AITL, nTFHL, or PTCL-NOS were analyzed.

**Figure S29. Estimated abundance of each immune cell fraction across the tumor microenvironment signature subtypes.** Adjusted p-value, *, **, ***, **** for < 0.05, 0.01, 0.001, 0.000, respectively.

**Figure S30. Correlation matrices for each immune cell. Bold black squares indicate clusters.**

Only significant correlations are plotted. Correlation coefficients are color-coded.


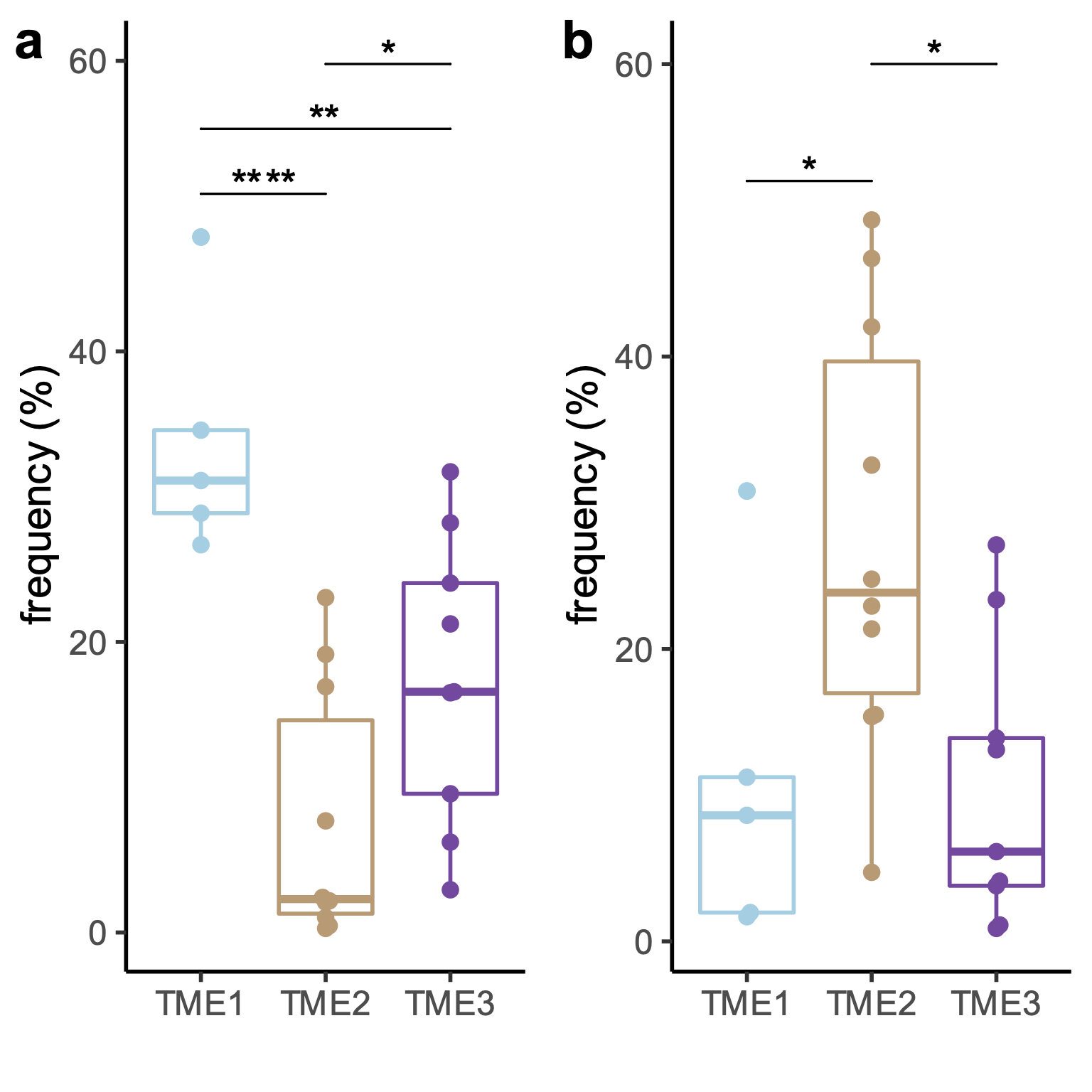


**Figure S31 Proportions of CD20 and CD163 positive cells assessed by immunohistochemistry across TME groups.**

1. CD20, (b) CD163.

**Figure S32. Pathological diagnoses across TME subtypes.**

**Figure S33. Gene set enrichment analysis between TME2 and TME3 PTCL-NOS.**

1. Analysis using hallmark gene sets. (b) Analysis using Reactome gene sets. FDR.q.val, false discovery rate q value.

**Figure S34. Hazard ratios for overall survival in each TME subtype.**

(a)

(b)

**Figure S35. Receiver operating characteristics of somatic mutation classifier.**

1. test1, using a randomly split 25% dataset of mutations from 15 paired samples. (b) test2, using mutations from three paired samples that were not used to develop the model.

**Figure S36. Importance of variables in the constructed prediction model of somatic mutations**


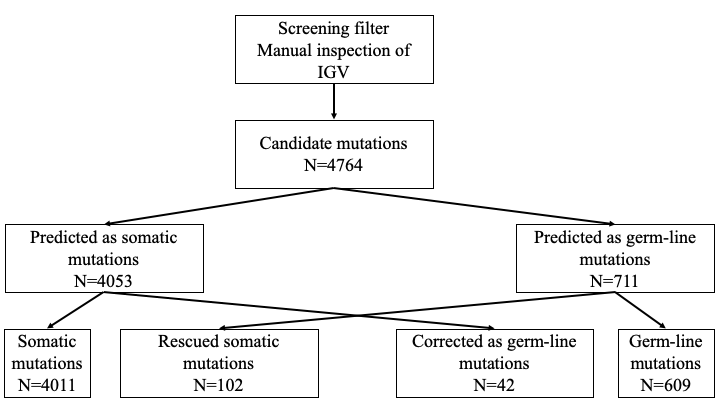


**Figure S37. Classification of candidate mutations by prediction model and manual inspection**


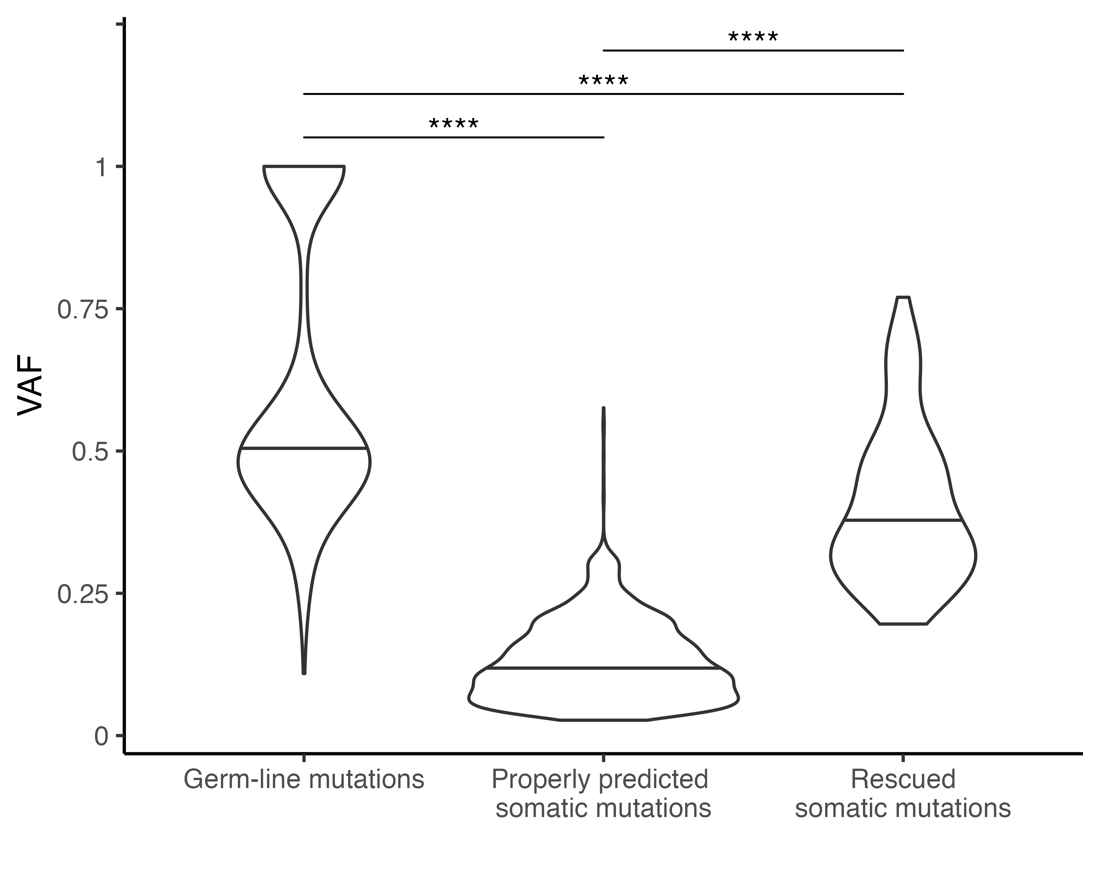


**Figure S38. Variant allele frequencies across mutation types.**

****, p-value < 0.0001.


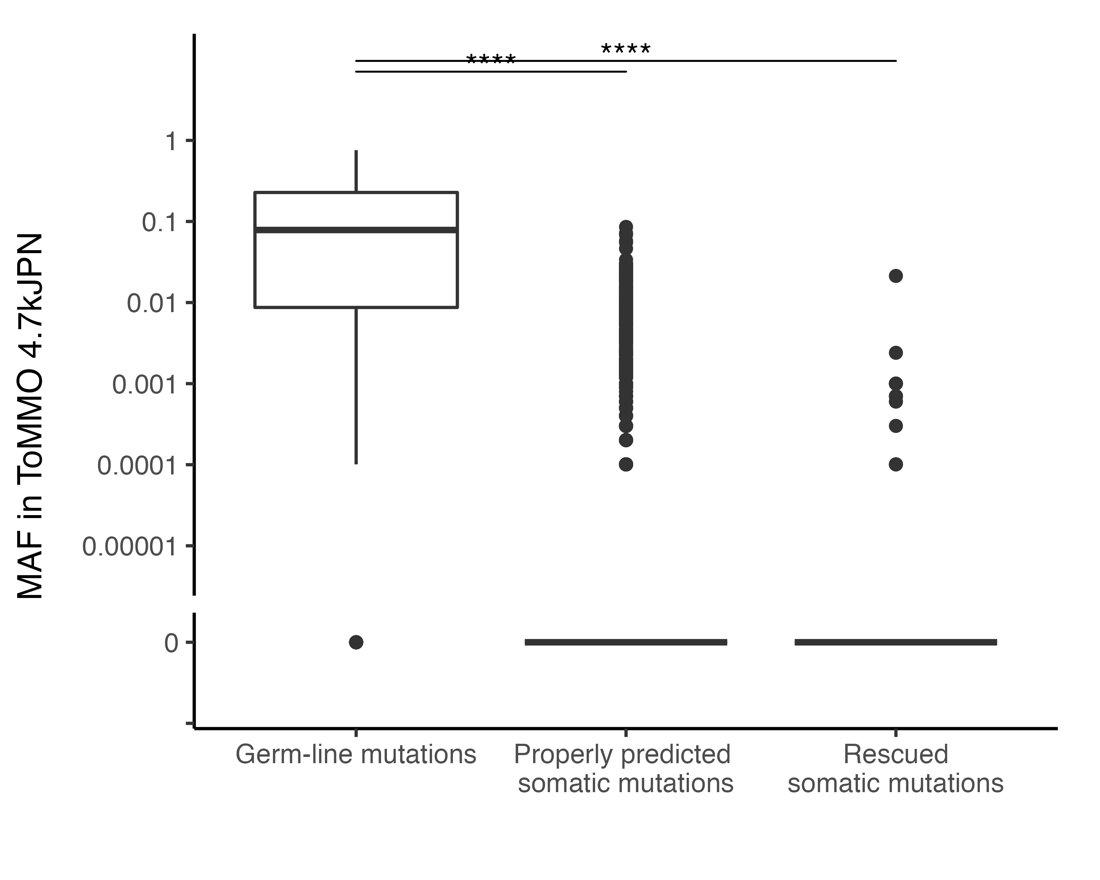


**Figure S39. Minor allele frequencies in ToMMo 4.7kJPN across mutation types.**

****, p-value < 0.0001. MAF, minor allele frequencies


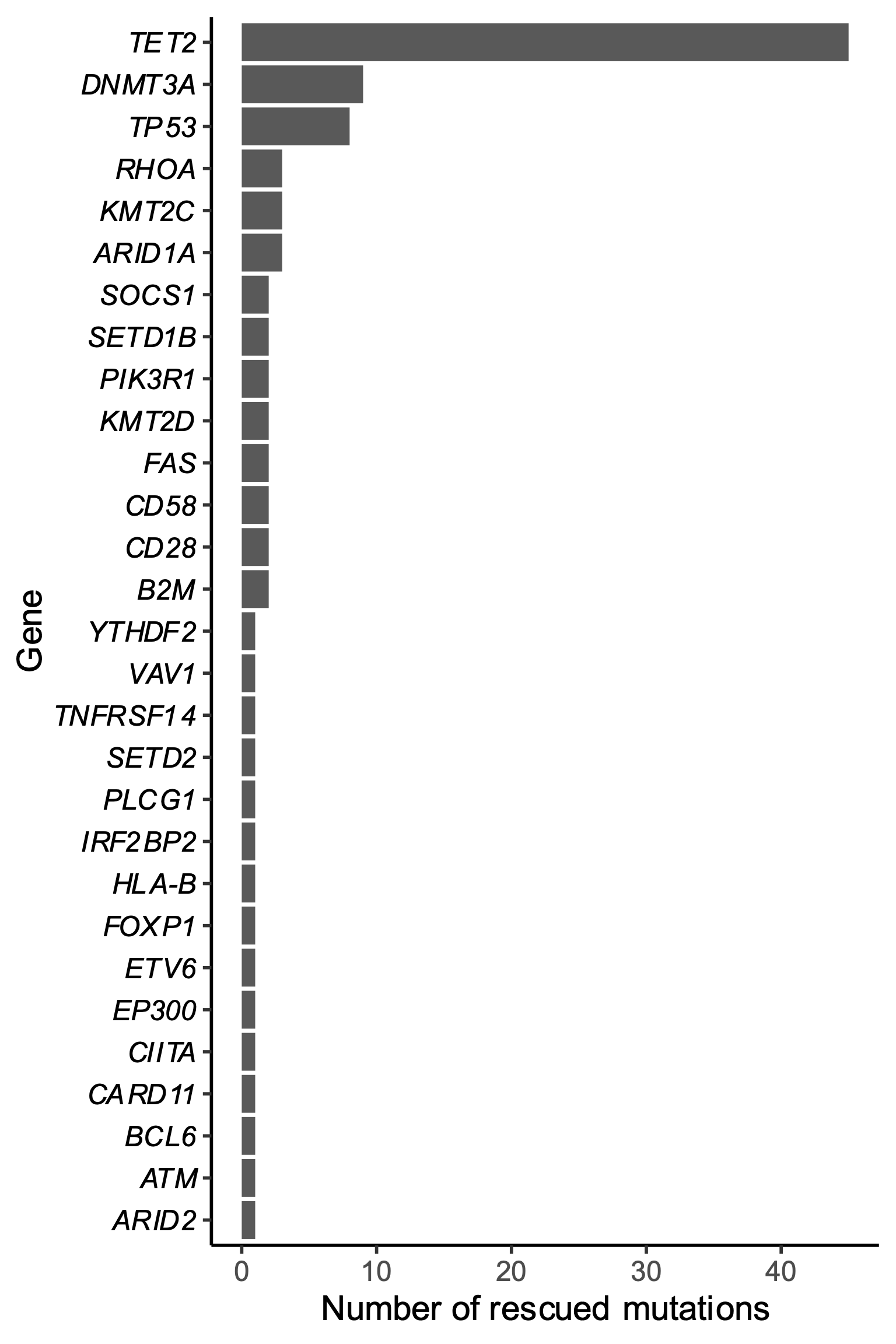


**Figure S40. Number of rescued somatic mutations per genes**

**Figure S41. The number of segments in the segmented copy ratio models between fresh frozen and formalin-fixed paraffin-embedded specimens.** FF, freshly frozen; FFPE, formalin-fixed paraffin-embedded; ****, p-value < 0.0001.

**Figure S42. Frequency of focal copy number alterations between the specimen types.**

FF, freshly frozen; FFPE, formalin-fixed paraffin-embedded; ns, not significant.


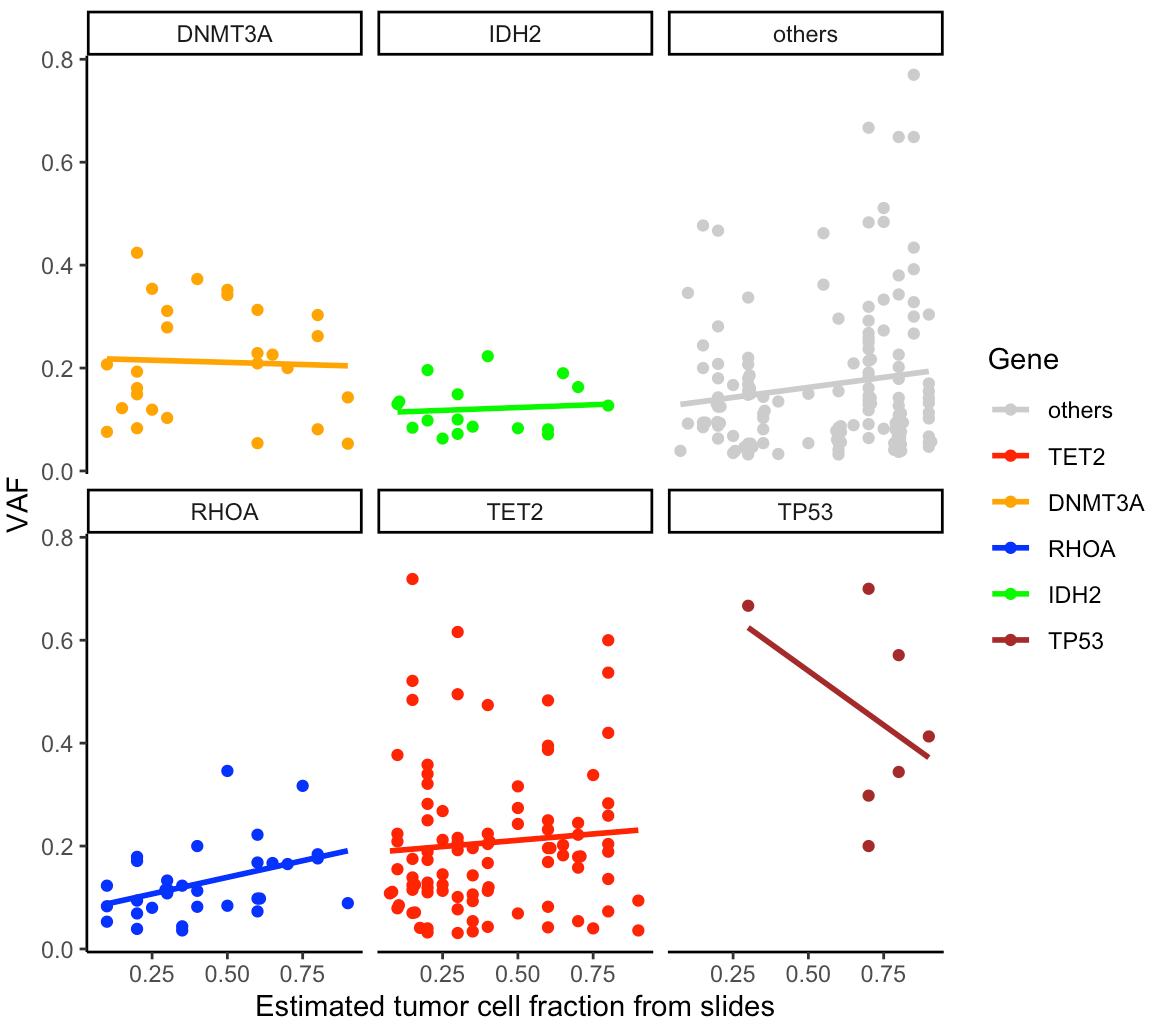


**Figure S43. Comparison of VAF of driver genes and estimated tumor cell fraction from slides**

Data points represent individual mutations, and a linear regression line is fitted for each gene.
